# Supplementary material for: Artificial Intelligence That Predicts Sensitizing Potential of Cosmetic Ingredients with Accuracy Comparable to Animal and In Vitro Tests—How Does the Infotechnomics Compare to Other “Omics” in the Cosmetics Safety Assessment?
Source: Int J Mol Sci. 2023 Apr 6;24(7):6801. doi: 10.3390/ijms24076801 (PMC10094956; doi:10.3390/ijms24076801)
Supplement: Supplementary file 1 [file ijms-24-06801-s001.zip › ijms-2280997-supplementary.pdf]

Table S1. Summary of patch test studies in patients with ACD.

Name of ingredient	Concentration (%)	Base	Occlusion (h)	Time to evaluate reactions	Application system	N	n	n (%)	Source
**Alpha-isomethylionone**	10	Pet.	48	48	Finn Chambers on Scanpor tape	422	9	2.10	[1]
**Ammylcinnamyl alcohol**	2	Pet.	48	48, 72, 96, 168	Finn Chambers (8 mm), applied on the back with Scanpor tape	1503	1	0.10	[2]
	2	Pet.	48	48, 96	Finn Chambers applied on the back	312	1	0.30	[3]
	2	Pet.	48	48, 72, 96, 168	Finn Chambers (8 mm), applied on the upper aspect of the back	254	1	0.40	[4]
**Amylcinnamaldehyde**	2	Pet.	48	n/a	TRUE Test and Finn Chambers	3119	3	0.10	[5]
	1	Pet.	48	48, 72, 96, 168	Finn Chambers (8 mm), applied on the back with Scanpor tape	1503	3	0.20	[2]
	2	Pet.	48	48, 96	Finn Chambers applied on the back	312	1	0.30	[3]
	2	Pet.	48	48, 96	Finn Chambers applied on Scanpor tape to the upper back of patients	308	2	0.60	[6]
	5	Pet.	48	48, 120	Finn Chambers secured to the skin with Scanpor tape	178	4	2.30	[7]

					applied to the upper back				
	2	Pet.	48	48, 72, 96	Van der Bend patch test chambers applied on the back with Micropore and fixed with Fixomull and Mefix	940	30	3.20	[8]
	2	Pet.	48	n/a	n/a	59	2	3.40	[9]
	1	Pet.	48	72, 96	n/a	405	18	4.40	[10]
**Anise alcohol**	1	Pet.	24, 36	72	n/a	2004	1	0.05	[11]
	1	Pet.	48	48, 72, 96, 168	Finn Chambers (8 mm), applied on the back with Scanpor tape	1503	1	0.10	[2]
	1	Pet.	48	72, 96	n/a	986	1	0.10	[12]
	1	Pet.	48	48, 96	Finn Chambers applied on Scanpor tape to the upper back of patients	308	2	0.60	[6]
	5	Pet.	48	48, 120	Finn Chambers (8 mm), applied on the upper back with Scanpor tape	167	3	1.80	[13]
**Benzalkonium chloride**	0.1	Aq.	48	48, 96	Komory IQ Chambers Chemotechnique Diagnostics	405	2	0.49	[14]
	0.1	Pet.	48	72, 96	Curatest Lohman&Rauscher, Rengsdorf Germany	1927	13	0.67	[15]
	0.1	Pet.	48	48, 96	n/a	385	5	1.30	[16]

	0.1	Aq.	48	48, 96	Finn Chambers applied on Scanpor tape to the upper back of patients	308	5	1.62	[6]
	0.1	Pet	24, 48	72	Finn Chambers (8 mm) on Scan-por (19 departments), Leukotest, Hal, Curatest, Haye, Intradex Service BV, Alphen aan den Rijn,	11 308	207	1.83	[17]
	0.1	Pet.	24, 48	72, 96	n/a	893	17	1.90	[18]
	0.1	Aq.	48	48, 72, 96, 168	Finn Chambers (8 mm), applied on the upper back with Scanpor tape	4892	210	4.29	[19]
	0.1	Aq.	48	48, 72, 96, 168	Finn Chambers (8 mm), applied on the upper back with Scanpor tape	686	38	5.50	[19]
	0.1	Aq.	48	48, 72	Finn Chambers applied on the upper back	2295	126	5.49	[20]
	0.1	Aq.	48	72, 120	Finn Chambers on Scanpor	34	2	5.88	[21]
	0.1	Aq.	48	48, 72, 96, 168	Finn Chambers (8 mm), applied on the upper aspect of the back	3838	318	8.30	[21]
	0.1	Aq.	48	48, 72, 96, 168	Finn Chambers (8 mm), applied on the upper aspect of the back	2979	262	8.80	[21]

	0.1	Aq.	48	48, 72, 96, 168	Finn Chambers (8 mm), applied on the upper aspect of the back	928	99	10.70	[4]
	0.1	Pet.	48	48, 96	Finn Chambers (8 mm), applied on the upper back with Scanpor tape	584	71	12.16	[22]
**Benzocaine**	5	Pet.	48	48, 96	Finn Chambers on Scanpor tape applied on back	125	0	0	[23]
	5	Pet.	48	48, 72, 96, 168	Finn Chambers (8 mm), applied on the upper aspect of the back	3086	65	2.10	[21]
	5	Pet.	48	48, 72, 96, 168	n/a	1526	3	0.20	[24]
	5	Pet.	48	48, 72, 96, 168	n/a	692	2	0.30	[25]
	5	Pet.	48	48, 72, 96, 168	n/a	772	2	0.30	[25]
	5	Pet.	48	48, 72	Aluminium patch test chambers mounted on Micropore carried out on the upper back	200	5	2.50	[26]
	5	Pet.	48	48, 72	Finn Chambers on Scanpor tape applied to upper back	2758	20	0.70	[27]
**Benzoic acid**	5	Pet.	48	72, 120	Finn Chambers on Scanpor	40	5	1.25	[21]

	5	Pet.	48	48, 72, 96, 168	Finn Chambers (8 mm), applied on the upper back with Scanpor tape	1963	96	4.90	[19]
	5	Pet.	48	48, 72, 96, 168	Finn Chambers (8 mm), applied on the upper aspect of the back	1963	96	4.90	[21]
	5	Pet.	48	48, 72, 96, 168	Finn Chambers (8 mm), applied on the upper aspect of the back	3077	175	5.70	[21]
	5	Pet.	48	48, 72, 96, 168	Finn Chambers on Scanpor tape applied to a patient's upper back	864	53	6.10	[4]
**Benzyl alcohol**	1	Pet.	48	72, 96	n/a	6993	22	0.30	[10]
	1	Pet.	24, 48	72	n/a	2166	7	0.30	[11]
	1	Pet.	48	48, 72, 96, 168	Finn Chambers on Scanpor tape applied to a patient's upper back	869	3	0.30	[4]
	1	Pet.	48	72, 96	n/a	79 770	258	0.32	[28]
	1	Pet.	48	48, 96	Finn Chambers on Scanpor tape to the upper back	4552	18	0.40	[29]
	1	Pet.	24, 48	72	Finn Chambers (8 mm) on Scan-por (19 departments), Leukotest, Hal, Curatest, Haye,	11 373	46	0.40	[17]

					Intradex Service BV, Alphen aan den Rijn,				
	5	pet.	48	48, 72, 120	Finn Chambers applied to the upper back	167	2	1.20	[13]
**Benzyl benzoate**	1	Pet.	24, 48	72	n/a	2003	1	0.05	[11]
	1	Pet.	48	48, 96	Finn Chambers applied on the back	312	1	0.30	[3]
	1	Pet.	48	48, 96	Finn Chambers applied on Scanpor tape to the upper back of patients	308	2	0.60	[6]
**Benzyl cinnamate**	5	Pet.	48	48, 72, 96, 168	Finn Chambers (8 mm), applied on the back with Scanpor tape	1503	1	0.10	[2]
	5	Pet.	48	72, 96	n/a	2872	4	0.14	[30]
	5	Pet.	24, 48	72	n/a	2042	6	0.30	[11]
	5	Pet.	48	48, 96	Finn Chambers applied on the back	312	1	0.30	[3]
	5	Pet.	48	48, 96	Finn Chambers applied on Scanpor tape to the upper back of patients	308	2	0.60	[6]
	5	Pet.	48	48,96	Finn Chambers applied on Scanpor tape to the upper back of patients	308	2	0.60	[6]
	5	Pet.	48	48, 120	Finn Chambers (8 mm), applied on the	167	8	4.80	[13]

					upper back with Scanpor tape				
	2	Pet.	48	n/a	n/a	59	4	6.80	[9]
**Benzyl salicylate**	1	Pet.	48	72, 96	n/a	3775	2	0.05	[30]
	1	Pet.	24, 48	72	n/a	2041	2	0.10	[11]
	1	Pet.	48	48, 72, 96, 168	Finn Chambers (8 mm), applied on the back with Scanpor tape	1503	3	0.20	[2]
	2	Pet.	48	48, 96	Finn Chambers applied on the back	312	4	1.30	[3]
	2	Pet.	48	48, 96, 168	Finn Chambers fixed with Scanpor	86	2	2.30	[31]
	2	Pet.	48	48, 120	Finn Chambers (8 mm), applied on the upper back with Scanpor tape	167	5	3.00	[13]
**Black rubber mix**	0.6	Pet.	48	72, 144, 168	IQ Chambers from Chemotechnique Diagnostics AB applied on the back	3112	16	0.50	[32]
	0.6	Pet.	48	72, 144, 168	IQ Chambers from Chemotechnique Diagnostics AB applied on the back	3825	23	0.60	[32]
	0.6	Pet.	48	48, 72, 96, 168	Finn Chambers (8 mm), applied on the upper aspect of the back	3092	25	0.80	[21]
	0.6	Pet.	48	48, 72, 96, 168	Finn Chambers (8 mm), applied on the	3840	35	0.90	[19]

					upper back with Scanpor tape				
	0.6	Pet.	48	48, 72	Finn Chambers on Sanpor applied on the upper back	373	5	1.30	[33]
	0.6	Pet.	48	48, 96	IQ Chambers	327	7	2.10	[34]
**Bronopol**	0.25	Pet.	48	48, 96	Finn Chambers fixed with Scanpor tape	1192	2	0.16	[35]
	0.25	Pet.	48	48, 96	Komory IQ Chambers Chemotechnique Diagnostics	405	1	0.20	[14]
	0.5	Pet.	48	48, 72, 96, 168	n/a	1389	4	0.30	[35]
	0.5	Pet.	48	48, 72, 96, 168	n/a	1394	6	0.40	[35]
	0.5	Pet.	48	72, 96	n/a	702	4	0.40	[30]
	0.5	Pet.	48	48, 72, 96	Finn Chambers (8 mm) on Scanpor tape	8149	38	0.47	[36]
	0.5	Pet.	48	72, 96	n/a	2138	11	0.50	[37]
	0.5	Pet.	48	48, 72, 96, 168	n/a	1457	7	0.50	[25]
	0.5	Pet.	48	48, 72, 96, 168	Finn Chambers (8mm) on Scanpor tape	11 272	67	0.60	[37]
	0.25	Pet.	48	48, 72, 96, 168	Finn Chambers (8 mm), applied on the upper aspect of the back	944	7	0.70	[21]
	0.5	Pet.	48	48, 96	Finn Chambers secured with Scanpor	3062	25	0.80	[39]

				tape and left on the back				
1	Aq.	48	48, 96	Finn Chambers apllied with Scanpor tape to the upper back	2298	20	0.80	[40]
0.5	Pet.	48	72, 96	n/a	1904	15	0.80	[30]
0.25	Pet.	48	48, 96	Finn Chambers on Scanpor tape to the upper back	4756	43	0.90	[29]
0.25	Pet.	48	48, 96	IQ Chambers	327	3	0.90	[34]
0.5	Pet.	48	72, 96	n/a	14 728	147	1.00	[10]
0.5	Pet.	48	48, 72	Finn Chambers on Scanpor tape applied to upper back	2758	27	1.00	[27]
0.5	Pet.	48	72, 96	n/a	10 124	120	1.20	[10]
0.5	Pet.	48	48, 72	Finn Chambers applied on the upper back	2295	28	1.20	[20]
0.5	Pet.	24, 48	72	Finn Chambers (8 mm) on Scan-por (19 departments), Leukotest, Hal, Curatest, Haye, Intradex Service BV, Alphen aan den Rijn,	11 443	134	1.20	[17]
0.5	Pet.	48	72, 96	n/a	6792	91	1.30	[10]
0.25	Pet.	48	48, 96	Finn Chambers (8 mm) or IQ Chambers	9361	122	1.30	[41]
0.5	Pet.	48	72, 96	n/a	42 882	613	1.40	[28]
0.5	Pet.	48	72, 96	n/a	2248	39	1.70	[37]

	0.5	Pet.	48	48, 72, 96, 168	n/a	1459	26	1.80	[25]
	0.5	Pet.	48	72, 96	n/a	1984	39	1.97	[37]
	0.25	Pet.	48	48, 72, 96, 168	Finn Chambers (8 mm), applied on the upper back with Scanpor tape	991	20	2.00	[19]
	0.25	Pet.	48	48, 72, 96, 168	Finn Chambers (8 mm), applied on the upper back with Scanpor tape	3841	81	2.10	[19]
	0.5	Pet.	48	48, 72, 96, 168	Finn Chambers (8 mm), applied on the upper back with Scanpor tape	328	7	2.10	[19]
	0.5	Pet.	48	72, 96	Curatest Lohman&Rauscher, Rengsdorf Germany	1927	48	2.50	[15]
	0.5	Pet.	48	48, 72, 96, 168	Finn Chambers (8 mm), applied on the upper aspect of the back	2142	54	2.50	[21]
	0.5	Pet.	48	48, 72, 96, 168	Finn Chambers (8 mm), applied on the upper back with Scanpor tape	4897	162	3.30	[19]
	0.25	Pet.	48	48, 96	Finn Chambers (8 mm), applied on the upper back with Scanpor tape	584	19	3.30	[22]
	0.5	Pet.	48	48, 72, 96, 168	Finn Chambers (8 mm), applied on the	4435	151	3.40	[21]

					upper aspect of the back				
	0.5	Pet.	48	48, 96	Finn Chambers on Scanpor tape applied to the areas of the back	342	16	5.00	[42]
**Butylhydroxyanisole (BHA)**	2	Pet.	48	48, 72, 96, 168	Finn Chambers (8 mm), applied on the upper aspect of the back	944	6	0.60	[4]
	2	Pet.	48	48, 96	Finn Chambers on Scanpor applied to the upper back	91	2	2.20	[43]
**Butylphenyl Methylpropional**	10	Pet.	48	48, 72, 96, 168	Finn Chambers (8 mm), applied on the back with Scanpor tape	1503	4	0.30	[2]
	10	Pet.	24, 48	72	n/a	2004	8	0.40	[11]
	10	Pet.	48	48, 96	Finn Chambers applied on Scanpor tape to the upper back of patients	308	2	0.60	[6]
	10	Pet.	48	72, 96	n/a	1003	6	0.60	[12]
	10	Pet.	48	48, 96	Finn Chambers applied on the back	312	3	1.00	[3]
	5	Pet.	48	48, 120	Finn Chambers (8 mm), applied on the upper back with Scanpor tape	167	3	1.80	[13]
**Caine Mix**	7	Pet.	48	72, 144, 168	IQ Chambers from Chemotechnique Diagnostics AB applied on the back	3112	22	0.70	[32]

	7	Pet.	48	72	Finn Chambers and TRUE Test applied on the back	3662	40	1.10	[32]
	7	Pet.	48	48, 72	Finn Chambers on Sanpor applied on the upper back	373	5	1.30	[33]
	7	Pet.	48	48, 96	n/a	385	5	1.30	[16]
	7	Pet.	48	48, 72, 96, 168	Finn Chambers (8 mm), applied on the upper back with Scanpor tape	1669	75	4.50	[19]
**Carba Mix**	3	Pet.	48	48, 96	Finn Chambers on Scanpor tape applied on back	125	1	0.80	[23]
	3	Pet.	48	48, 72	Finn Chambers on Sanpor applied on the upper back	244	3	1.20	[33]
	3	Pet.	48	48, 72, 96, 168	Finn Chambers (8 mm), applied on the upper back with Scanpor tape	3841	177	4.60	[19]
	3	Pet.	48	48, 72, 96, 168	Finn Chambers (8 mm), applied on the upper aspect of the back	3085	176	5.70	[21]
	3	Pet.	48	96	Finn Chambers applied to the back of the patients	618	45	7.30	[44]
**Cinnamal**	1	Pet.	48	48, 72, 96, 168	Finn Chambers (8 mm), applied on the back with Scanpor tape	1503	20	1.30	[2]

	1	Pet.	48	48	Finn Chambers on Scanpor tape	422	7	1.70	[1]
	1	Pet.	48	48, 96	Finn Chambers applied on the back	312	28	9.00	[3]
**Cinnamic alcohol**	2	Pet.	48	n/a	TRUE Test and Finn Chambers	3119	10	0.30	[5]
	1	Pet.	24, 48	72	n/a	2063	13	0.60	[11]
	1	Pet.	48	48, 72, 96, 168	Finn Chambers (8 mm), applied on the back with Scanpor tape	1501	10	0.70	[2]
	1	Pet.	48	72, 96	n/a	5650	46	0.80	[12]
	1	Pet.	48	48	Finn Chambers on Scanpor tape	422	13	3.10	[1]
	5	lanolina	48	48, 120	Finn Chambers (8 mm), applied on the upper back with Scanpor tape	167	11	6.60	[13]
	1	Pet.	48	48, 96	Finn Chambers on Scanpor applied to the upper back	40	4	10.00	[43]
	1	Pet.	48	72, 96	n/a	405	45	11.10	[10]
	1	Pet.	48	48, 96	Finn Chambers applied on the back	312	35	11.20	[3]
	2	Pet.	48	48, 72, 96	Van der Bend patch test chambers applied on the back with Micropore and fixed with Fixomull and Mefix	940	129	13.70	[8]
	2	Pet.	48	48, 96, 168	Finn Chambers fixed with Scanpor	86	12	13.90	[31]
	1	Pet.	48	n/a	n/a	59	27	45.80	[9]

**Cinnamic aldehyde**	1	Pet.	48	48, 96	Finn Chambers applied on Scanpor tape to the upper back of patients	308	2	0.60	[6]
	1	Pet.	24, 48	72	n/a	2063	21	1.00	[11]
	1	Pet.	48	48, 72, 96, 168	Finn Chambers (8 mm), applied on the upper back with Scanpor tape	1321	22	1.70	[19]
	1	Pet.	48	48, 72, 96, 168	Finn Chambers (8 mm), applied on the upper aspect of the back	3085	56	1.80	[21]
	1	Pet.	48	48, 72, 96, 168	Finn Chambers (8 mm), applied on the upper aspect of the back	943	17	1.80	[4]
	1	Pet.	48	48, 72, 96, 168	Finn Chambers (8 mm), applied on the upper back with Scanpor tape	3114	62	2.00	[19]
	1	Pet.	48	48, 72, 96, 168	Finn Chambers (8 mm), applied on the upper aspect of the back	3114	62	2.00	[21]
	1	Pet.	48	48, 72, 96, 168	Finn Chambers (8 mm), applied on the upper aspect of the back	4435	137	3.10	[21]
	1	Pet.	48	72, 120	Finn Chambers on Scanpor	34	2	6.00	[21]

	1	Pet.	48	48, 96	Finn Chambers on Scanpor applied to the upper back	40	5	12.50	[43]
	1	Pet.	48	48, 120	Finn Chambers (8 mm), applied on the upper back with Scanpor tape	167	24	14.40	[13]
	1	Pet.	48	n/a	n/a	59	21	35.60	[9]
**Citral**	2	Pet.	48	n/a	TRUE Test and Finn Chambers	3119	6	0.20	[5]
	2	Pet.	48	48, 72, 96, 168	Finn Chambers (8 mm), applied on the back with Scanpor tape	1502	4	0.30	[2]
	2	Pet.	48	48, 96	Finn Chambers applied on Scanpor tape to the upper back of patients	308	2	0.60	[6]
	2	Pet.	48	48	Finn Chambers on Scanpor tape	422	5	1.20	[1]
	2	Pet.	48	48, 96, 168	Finn Chambers fixed with Scanpor	86	2	2.30	[31]
	2	Pet.	48	48, 72, 96	Van der Bend patch test chambers applied on the back with Micropore and fixed with Fixomull and Mefix	205	27	13.20	[8]
	2	Pet.	48	72, 96	n/a	137	26	19.00	[10]
	2	Pet.	48	72	n/a	2021	13	0.60	[11]
	2	Pet.	48	48, 96	Finn Chambers applied on the back	312	10	3.20	[3]

**Citronellol**	1	Pet.	48	n/a	TRUE Test and Finn Chambers	3119	3	0.10	[5]
	1	Pet.	48	48, 72, 96, 168	Finn Chambers (8 mm), applied on the back with Scanpor tape	1503	1	0.10	[2]
	1	Pet.	24, 48	72	n/a	2003	9	0.40	[11]
	1	Pet.	48	48, 96	Finn Chambers applied on Scanpor tape to the upper back of patients	308	2	0.60	[6]
	1	Pet.	48	48, 96	Finn Chambers applied on the back	312	3	1.00	[3]
	1	Pet.	48	48, 72, 96	Van der Bend patch test chambers applied on the back with Micropore and fixed with Fixomull and Mefix	205	11	5.40	[8]
	5	Pet.	48	48, 120	Finn Chambers secured to the skin with Scanpor tape applied to the upper back	178	10	5.60	[7]
	1	Pet.	48	72, 96	n/a	137	8	5.80	[10]
	5	Pet.	48	48, 120	n/a	218	19	8.70	[44]
**Cobalt (di)chloride**	1	Pet.	48	48, 96	n/a	50	2	4.00	[16]
	1	Pet.	48	48, 96	IQ Chambers	327	23	7.00	[34]
	1	Pet.	48	48, 72	Aluminium patch test chambers mounted on Micropore carried	200	16	8.00	[26]

				out on the upper back				
	1	Pet.	48	48, 96	n/a	385	35	9.00	[16]
	1	Pet.	48	48, 72, 96, 168	Finn Chambers (8 mm), applied on the upper back with Scanpor tape	3818	393	10.30	[19]
	1	Pet.	48	48, 72, 96, 168	n/a	1471	50	3.40	[25]
	1	Pet.	48	72, 144, 168	IQ Chambers from Chemotechnique Diagnostics AB applied on the back	3825	245	6.40	[32]
	1	Pet.	48	72, 144, 168	IQ Chambers from Chemotechnique Diagnostics AB applied on the back	3112	140	4.50	[32]
	1	Pet.	48	96	Finn Chambers applied to the back of the patients	618	65	10.50	[44]
	1	Pet.	48	72	Finn Chambers and TRUE Test applied on the back	3662	42	6.60	[32]
	1	Pet.	48	48, 72, 96, 168	Finn Chambers (8 mm), applied on the upper aspect of the back	3092	359	11.60	[21]
	1	Pet.	48	48, 72, 96, 168	n/a	1399	82	5.90	[35]
	1	Pet.	48	48, 96	Finn Chambers on Scanpor tape applied on back	125	5	4.00	[23]

	1	Pet.	48	72, 120	Finn Chambers on Scanpor	40	4	10.00	[21]
	1	Pet.	48	48, 72	Finn Chambers on Sanpor applied on the upper back	373	24	6.40	[33]
	1	Pet.	48	72, 96	n/a	10 124	494	4.90	[10]
	1	Pet.	48	72, 96	n/a	14 728	1016	6.90	[10]
**Cocamidopropyl betaine**	1	Aq.	48	48, 96	Finn Chambers applied on Scanpor tape to the upper back of patients	308	2	0.60	[6]
	1	Aq.	48	72, 96	n/a	1903	37	1.90	[30]
	1	Aq.	48	72, 96	n/a	1723	41	2.40	[37]
	1	Aq.	48	72, 96	n/a	744	22	3.00	[30]
	1	Aq.	48	48, 72, 96, 168	Finn Chambers (8 mm), applied on the upper aspect of the back	935	32	3.40	[4]
	1	Aq.	48	48, 72, 96, 168	Finn Chambers (8 mm), applied on the upper aspect of the back	3003	120	4.00	[21]
	1	Aq.	48	48, 96	Aluminium chambers carried out on the upper back	33	2	6.00	[45]
**Colophonium**	20	Pet.	48	48, 96	Finn Chambers on Scanpor tape applied on back	125	1	0.80	[23]
	20	Pet.	48	48, 72, 96, 168	Finn Chambers (8 mm), applied on the	4447	98	2.20	[21]

				upper aspect of the back				
	20	Pet.	48	120, 168	IQ Ultra Chambers applied on the patient's back	1608	38	2.40	[46]
	20	Pet.	48	72, 144, 168	IQ Chambers from Chemotechnique Diagnostics AB applied on the back	3112	75	2.40	[32]
	20	Pet.	48	72, 120	Finn Chambers on Scanpor	40	1	2.50	[21]
	20	Pet.	48	48, 72, 96, 168	Finn Chambers (8 mm), applied on the upper aspect of the back	942	24	2.50	[4]
	20	Pet.	48	48, 72, 96, 168	Finn Chambers (8 mm), applied on the upper back with Scanpor tape	1322	33	2.50	[19]
	20	Pet.	48	48, 72, 96, 168	Finn Chambers (8 mm), applied on the upper aspect of the back	3076	80	2.60	[21]
	20	Pet.	48	48, 72, 96, 168	Finn Chambers (8 mm), applied on the upper back with Scanpor tape	3842	100	2.60	[19]
	20	Pet.	48	48, 72, 96, 168	Finn Chambers (8 mm), applied on the upper back with Scanpor tape	4908	128	2.60	[19]

	20	Pet.	48	96	Finn Chambers applied to the back of the patients	618	17	2.80	[44]
	20	Pet.	48	48, 96	n/a	385	11	2.90	[16]
	20	Pet.	48	48, 72, 96, 168	n/a	1992	62	3.10	[24]
	20	Pet.	48	72, 96	n/a	967	32	3.30	[18]
	20	Pet.	48	72, 96	n/a	2248	76	3.40	[37]
	20	Pet.	48	72, 144, 168	IQ Chambers from Chemotechnique Diagnostics AB applied on the back	3825	130	3.40	[32]
	20	Pet.	48	48, 72	Finn Chambers on Scanpor tape applied to upper back	2758	99	3.60	[27]
	20	Pet.	48	72, 96	n/a	10 124	389	3.80	[10]
	20	Pet.	48	72, 96	n/a	14 728	574	3.90	[10]
	20	Pet.	48	72, 96	n/a	2046	80	3.90	[37]
	20	Pet.	48	72, 96	n/a	37 011	1603	4.33	[12]
	20	Pet.	48	48, 72, 96, 168	n/a	1468	64	4.40	[25]
	20	Pet.	48	48, 96	IQ Chambers	327	15	4.60	[34]
	20	Pet.	48	72, 168	Finn Chambers on Scanpor tape	2598	119	4.60	[47]
	20	Pet.	48	48, 72 96, 168	n/a	1399	64	4.60	[35]
	20	Pet.	48	48, 72	Finn Chambers on Sanpor applied on the upper back	373	18	4.80	[33]
	20	Pet.	48	48, 96	Finn Chambers secured with Scanpor	3062	159	5.20	[38]

					tape and left on the back				
	20	Pet.	48	72	Finn Chambers and TRUE Test applied on the back	3662	198	5.40	[32]
	20	Pet.	48	48, 72	Aluminium patch test chambers mounted on Micropore carried out on the upper back	200	11	5.50	[26]
	20	Pet.	48	48, 96	Finn Chambers on Scanpor applied to the upper back	91	15	16.50	[43]
**Coumarin**	5	Pet.	48	n/a	TRUE Test and Finn Chambers	3119	3	0.10	[5]
	5	Pet.	48	48, 72, 96, 168	Finn Chambers (8 mm), applied on the back with Scanpor tape	1503	3	0.20	[2]
	5	Pet.	48	48, 96	Finn Chambers applied on the back	312	1	0.30	[3]
	5	Pet.	24, 48	72	n/a	2020	8	0.40	[11]
	5	Pet.	48	48, 96	Finn Chambers applied on Scanpor tape to the upper back of patients	308	2	0.60	[6]
	5	Pet.	48	48, 96, 168	Finn Chambers fixed with Scanpor	86	1	1.20	[31]
	5	Pet.	48	48, 120	Finn Chambers (8 mm), applied on the upper back with Scanpor tape	167	2	1.20	[13]

	5	Pet.	48	48, 72, 96	Van der Bend patch test chambers applied on the back with Micropore and fixed with Fixomull and Mefix	205	9	4.40	[8]
	5	Pet.	48	72, 96	n/a	137	6	5.80	[10]
**Diazolidynyl urea**	2	Pet.	48	48, 96	Finn Chambers applied on Scanpor tape to the upper back of patients	308	1	0.30	[6]
	2	Pet.	48	72, 144, 168	IQ Chambers from Chemotechnique Diagnostics AB applied on the back	3112	16	0.50	[32]
	2	Pet.	48	48, 96	Komory IQ Chambers Chemotechnique Diagnostics	405	2	0.50	[14]
	2	Pet.	48	48, 96	Finn Chambers secured with Scanpor tape and left on the back	3062	21	0.70	[38]
	2	Pet.	48	72, 96	Curatest Lohman&Rauscher, Rengsdorf Germany	1927	13	0.70	[15]
	2	Pet.	48	72, 96	n/a	6979	48	0.70	[10]
	2	Pet.	48	72, 96	n/a	78 711	587	0.75	[28]
	2	Aq.	48	48, 96	Finn Chambers fixed with Scanpor tape	3900	31	0.79	[35]
	2	Pet.	48	48, 72, 96, 168	n/a	1398	15	1.10	[35]

	2	Pet.	48	48, 96	Finn Chambers (8 mm) or IQ Chambers	9362	103	1.10	[41]
	2	Pet.	24, 48	72	Finn Chambers (8 mm) on Scan-por (19 departments), Leukotest, Hal, Curatest, Haye, Intradex Service BV, Alphen aan den Rijn,	7812	98	1.25	[17]
	1	Aq.	48	48, 72, 96, 168	Finn Chambers (8 mm), applied on the upper aspect of the back	3086	40	1.30	[21]
	2	Pet.	48	72, 144, 168	IQ Chambers from Chemotecgnique Diagnostics AB applied on the back	3825	54	1.40	[32]
	2	Pet.	48	48, 96	Finn Chambers (8 mm), applied on the upper back with Scanpor tape	584	8	1.40	[22]
	2	Pet.	48	48, 72, 96, 168	Finn Chambers (8mm) on Scanpor tape	11 270	178	1.60	[38]
	2	Pet.	48	72	Finn Chambers and TRUE Test applied on the back	3662	66	1.90	[32]
	1	Aq.	48	48, 72, 96, 168	Finn Chambers on Scanpor tape applied to a patient’s upper back	940	19	2.00	[4]
	1	Aq.	48	48, 72, 96, 168	Finn Chambers (8 mm), applied on the	4433	98	2.20	[21]

					upper aspect of the back				
	1	Aq.	48	48, 72, 96, 168	Finn Chambers (8 mm), applied on the upper aspect of the back	3840	92	2.40	[21]
	1	Aq.	48	48, 72, 96, 168	Finn Chambers (8 mm), applied on the upper back with Scanpor tape	1319	33	2.50	[19]
	1	Pet.	48	48, 72, 96, 168	Finn Chambers (8 mm), applied on the upper aspect of the back	3091	77	2.50	[21]
	1	Pet.	48	48, 72, 96, 168	Finn Chambers (8 mm), applied on the upper back with Scanpor tape	1033	30	2.90	[19]
	1	Pet.	48	48, 72, 96, 168	Finn Chambers (8 mm), applied on the upper back with Scanpor tape	4897	152	3.10	[19]
	1	Pet.	48	48, 72, 96, 168	Finn Chambers on Scanpor tape applied to a patient's upper back	942	29	3.10	[4]
	1	Pet.	48	48, 72, 96, 168	Finn Chambers (8 mm), applied on the upper aspect of the back	3842	134	3.50	[21]
	1	Pet.	48	48, 72, 96, 168	Finn Chambers (8 mm), applied on the	4439	164	3.70	[21]

					upper aspect of the back				
	2	Pet.	48	48, 96	Finn Chambers on Scanpor tape applied to the areas of the back	342	13	3.80	[42]
**DMDM Hydantoin**	2	Pet.	24, 48	72	Finn Chambers (8 mm) on Scan-por (19 departments), Leukotest, Hal, Curatest, Haye, Intradex Service BV, Alphen aan den Rijn,	1374	4	0.30	[17]
	1	Aq.	48	48, 72, 96, 168	Finn Chambers (8 mm), applied on the upper back with Scanpor tape	1042	5	0.50	[19]
	2	Pet.	48	72, 96	n/a	78 266	388	0.50	[28]
	2	Aq.	48	48, 96	Finn Chambers applied on Scanpor tape to the upper back of patients	308	2	0.60	[6]
	2	Aq.	48	48, 96	Komory IQ Chambers Chemotechnique Diagnostics	405	3	0.70	[14]
	2	Aq.	48	48, 72, 96, 168	Finn Chambers (8mm) on Scanpor tape	1946	14	0.70	[38]
	1	Pet.	48	48, 72, 96, 168	Finn Chambers (8 mm), applied on the	1321	11	0.80	[19]

					upper back with Scanpor tape				
	2	Aq.	48	48, 96	Finn Chambers fixed with Scanpor tape	1163	10	0.85	[35]
	2	Aq.	48	72, 96	Curatest Lohman&Rauscher, Rengsdorf Germany	1927	17	0.90	[15]
	2	Aq.	48	48, 72, 96, 168	Finn Chambers (8 mm), applied on the upper back with Scanpor tape	411	5	1.20	[19]
	2	Aq.	48	48, 72, 96, 168	Finn Chambers (8 mm), applied on the upper aspect of the back	411	5	1.20	[21]
	1	Aq.	48	48, 72, 96, 168	Finn Chambers (8 mm), applied on the upper back with Scanpor tape	3428	44	1.30	[19]
	1	Aq.	48	48, 72, 96, 168	Finn Chambers (8 mm), applied on the upper aspect of the back	3428	45	1.30	[21]
	1	Aq.	48	48, 72, 96, 168	Finn Chambers (8 mm), applied on the upper aspect of the back	4433	62	1.40	[21]
	2	Aq.	48	48, 72, 96, 168	Finn Chambers (8 mm), applied on the upper aspect of the back	3085	43	1.40	[21]
	1	Pet.	48	48, 72, 96, 168	Finn Chambers on Scanpor tape applied	937	14	1.50	[4]

					to a patient's upper back				
	1	Aq.	48	48, 96	Finn Chambers (8 mm), applied on the upper back with Scanpor tape	584	10	1.70	[22]
	3	Aq.	48	48, 72	Finn Chambers applied on the upper back	2295	39	1.70	[20]
	2	Aq.	48	48, 72, 96, 168	Finn Chambers on Scanpor tape applied to a patient's upper back	327	6	1.80	[4]
	1	Pet.	48	48, 72, 96, 168	Finn Chambers (8 mm), applied on the upper aspect of the back	3087	62	2.00	[21]
	2	Aq.	48	48, 96	Finn Chambers on Scanpor tape to the upper back	3709	78	2.10	[29]
	1	Aq.	48	48, 72, 96, 168	Finn Chambers (8 mm), applied on the upper back with Scanpor tape	4897	108	2.20	[19]
	1	Pet.	48	48, 72, 96, 168	Finn Chambers (8 mm), applied on the upper aspect of the back	3757	83	2.20	[21]
	1	Pet.	48	48, 72, 96, 168	Finn Chambers (8 mm), applied on the	4439	115	2.60	[21]

					upper aspect of the back				
	1	Pet.	48	48, 72, 96, 168	Finn Chambers (8 mm), applied on the upper back with Scanpor tape	4897	137	2.80	[19]
	2	Aq.	48	48, 96	Finn Chambers on Scanpor tape applied to the areas of the back	342	14	4.00	[42]
**Epoxy resin**	1	Pet.	48	48, 96	Finn Chambers on Scanpor tape applied on back	125	0	0.00	[23]
	1	Pet.	48	72, 96	n/a	10 124	97	1.00	[10]
	1	Pet.	48	72, 168	Finn Chambers on Scanpor tape	2598	26	1.00	[47]
	1	Pet.	48	48, 72	Finn Chambers on Scanpor tape applied to upper back	2758	3	0.10	[27]
	1	Pet.	48	48, 72, 96, 168	n/a	1471	16	1.10	[25]
	1	Pet.	48	48, 72, 96, 168	n/a	1469	60	4.10	[25]
	1	Pet.	48	72, 144, 168	IQ Chambers from Chemotechnique Diagnostics AB applied on the back	3112	37	1.20	[32]
	1	Pet.	48	72, 144, 168	IQ Chambers from Chemotechnique Diagnostics AB applied on the back	3825	46	1.20	[32]

	1	Pet.	48	72	Finn Chambers and TRUE Test applied on the back	3662	48	1.30	[32]
	1	Pet.	48	48, 72, 96, 168	Finn Chambers (8 mm), applied on the upper aspect of the back	3089	43	1.40	[21]
	1	Pet.	48	96	Finn Chambers applied to the back of the patients	618	9	1.50	[44]
	1	Pet.	48	48, 72	Aluminium patch test chambers mounted on Micropore carried out on the upper back	200	3	1.50	[26]
	1	Pet.	48	48, 72, 96, 168	Finn Chambers (8 mm), applied on the upper back with Scanpor tape	3846	54	1.60	[19]
	1	Pet.	48	48, 72, 96, 168	n/a	1399	23	1.60	[35]
	1	Pet.	48	48, 96	n/a	385	6	1.60	[16]
	1	Pet.	48	48, 72	Finn Chambers on Sanpor applied on the upper back	373	3	0.80	[33]
**Ethylenediamine (dichloride)**	1	Pet.	48	48, 96	n/a	385	5	1.30	[16]
	1	Pet.	48	48, 96	Finn Chambers on Scanpor tape applied on back	125	3	2.40	[23]

	1	Pet.	48	48, 72	Finn Chambers on Sanpor applied on the upper back	373	24	6.40	[33]
	1	Pet.	48	48, 72, 96, 168	Finn Chambers (8 mm), applied on the upper aspect of the back	944	35	3.70	[4]
	1	Pet.	48	72, 144, 168	IQ Chambers from Chemotechnique Diagnostics AB applied on the back	3112	6	0.20	[32]
	1	Pet.	48	72, 144, 168	IQ Chambers from Chemotechnique Diagnostics AB applied on the back	3825	54	1.40	[32]
	1	Pet.	48	48, 96	Finn Chambers (8 mm), applied on the upper back with Scanpor tape	584	12	2.10	[22]
	1	Pet.	48	48, 72, 96, 168	Finn Chambers (8 mm), applied on the upper back with Scanpor tape	3843	92	2.40	[19]
	1	Pet.	48	48, 72, 96, 168	Finn Chambers (8 mm), applied on the upper aspect of the back	3090	77	2.50	[21]
	1	Pet.	48	48, 96	IQ Chambers	327	11	3.40	[34]
**Eugenol**	2	Pet.	48	n/a	TRUE Test and Finn Chambers	3119	6	0.20	[5]

	1	Pet.	48	48, 72, 96, 168	Finn Chambers (8 mm), applied on the back with Scanpor tape	1502	4	0.30	[2]
	1	Pet.	24, 48	72	n/a	2065	11	0.50	[11]
	1	Pet.	48	48, 96	Finn Chambers applied on the back	312	3	1.00	[3]
	2	Pet.	48	48	Finn Chambers on Scanpor tape	422	8	1.90	[1]
	2	Pet.	48	72, 120	Finn Chambers on Scanpor	32	1	3.10	[21]
	1	Pet.	48	48, 96	Finn Chambers on Scanpor applied to the upper back	40	2	5.00	[43]
	5	Pet.	48	48, 120	Finn Chambers (8 mm), applied on the upper back with Scanpor tape	167	13	7.80	[13]
	1	Pet.	48	72, 96	n/a	405	39	9.60	[10]
	2	Pet.	48	48, 72, 96	Van der Bend patch test chambers applied on the back with Micropore and fixed with Fixomull and Mefix	940	118	12.60	[8]
	2	Pet.	48	48, 96, 168	Finn Chambers fixed with Scanpor	86	12	13.90	[31]
	2	Pet.	48	n/a	n/a	59	19	32.20	[9]
**Evernia Furfuracea Extract**	1	Pet.	48	48, 96	Finn Chambers applied on the back	312	8	2.60	[3]
	1	Pet.	24, 48	72	n/a	1658	45	2.70	[11]
	1	Pet.	48	48, 72, 96, 168	Finn Chambers (8 mm), applied on the	1503	50	3.30	[2]

					back with Scanpor tape				
	1	Pet.	48	72, 96	n/a	1947	129	6.60	[12]
	1	Pet.	48	72, 96	n/a	319	23	7.20	[37]
**Evernia Prunastri Extract**	2	Pet.	48	n/a	TRUE Test and Finn Chambers	3119	16	0.50	[5]
	2	Pet.	48	48, 96	Finn Chambers applied on the back	312	3	1.00	[3]
	2	Pet.	48	48	Finn Chambers on Scanpor tape	422	6	1.40	[1]
	1	Pet.	48	48, 72, 96, 168	Finn Chambers (8 mm), applied on the back with Scanpor tape	1503	31	2.10	[2]
	1	Pet.	24, 48	72	n/a	2063	46	2.20	[11]
	2	Pet.	48	48, 96, 168	Finn Chambers fixed with Scanpor	86	2	2.30	[31]
	2	Pet.	48	72, 96	n/a	607	32	5.30	[37]
	5	Pet.	48	48, 120	Finn Chambers (8 mm), applied on the upper back with Scanpor tape	167	22	13.20	[13]
	2	Pet.	48	n/a	n/a	59	14	23.70	[9]
	2	Pet.	48	48, 72, 96	Van der Bend patch test chambers applied on the back with Micropore and fixed with Fixomull and Mefix	940	230	24.60	[8]
	2	Pet.	48	48, 96	Finn Chambers on Scanpor applied to the upper back	40	12	30.00	[43]

**Farnesol**	5	Pet.	48	48, 72, 96, 168	Finn Chambers (8 mm), applied on the back with Scanpor tape	1502	5	0.30	[2]
	5	Pet.	48	n/a	TRUE Test and Finn Chambers	3119	14	0.40	[5]
	5	Pet.	48	48, 96	Finn Chambers applied on Scanpor tape to the upper back of patients	308	2	0.60	[6]
	5	Pet.	48	72	n/a	4238	38	0.90	[11]
	5	Pet.	48	48, 96, 168	Finn Chambers fixed with Scanpor	86	1	1.20	[31]
	5	Pet.	48	48, 96	Finn Chambers applied on the back	312	5	1.60	[3]
	5	Pet.	48	48, 72, 96	Van der Bend patch test chambers applied on the back with Micropore and fixed with Fixomull and Mefix	205	27	13.20	[8]
	5	Pet.	48	72, 96	n/a	137	19	13.90	[10]
**Formaldehyde**	1	Aq.	48	72, 168	Finn Chambers on Scanpor tape	2598	5	0.20	[47]
	1	Aq.	48	72, 96	n/a	14 728	133	0.90	[10]
	1	Aq.	48	72, 96	Curatest Lohman&Rauscher, Rengsdorf Germany	1927	21	1.10	[15]
	1	Aq.	48	72, 96	n/a	10 124	104	1.10	[10]
	1	Aq.	48	48, 96	Finn Chambers applied on Scanpor	308	4	1.30	[6]

					tape to the upper back of patients				
	1	Aq.	48	72, 96	n/a	972	13	1.30	[18]
	1	Aq.	48	120, 168	IQ Ultra Chambers applied on the patient’s back	1608	21	1.30	[46]
	1	Aq.	48	72, 96	n/a	121 558	1908	1.60	[28]
	1	Aq.	48	48, 96	Komory IQ Chambers Chemotechnique Diagnostics	405	7	1.70	[14]
	1	Aq.	48	48, 96	Finn Chambers fixed with Scanpor tape	7838	135	1.70	[35]
	1	Aq.	48	72, 96	n/a	2248	40	1.80	[37]
	1	Aq.	48	48, 72, 96, 168	n/a	1397	27	1.90	[35]
	1	Aq.	48	48, 96	Finn Chambers (8 mm) or IQ Chambers	9347	178	1.90	[41]
	1	Pet.	48	48, 96	Finn Chambers secured with Scanpor tape and left on the back	3062	64	2.10	[38]
	1	Aq.	48	48, 72, 96, 168	n/a	1993	44	2.20	[24]
	1	Aq.	48	72, 96	n/a	27 907	609	2.20	[17]
	1	Aq.	48	72, 96	n/a	2047	48	2.30	[37]
	1	Aq.	48	72, 144, 168	IQ Chambers from Chemotechnique Diagnostics AB applied on the back	3112	78	2.50	[32]
	1	Aq.	48	72	Finn Chambers and TRUE Test applied on the back	3662	92	2.50	[32]

	1	Aq.	48	48, 72, 96, 168	n/a	1395	35	2.50	[35]
	1	Aq.	48	72, 144, 168	IQ Chambers from Chemotechnique Diagnostics AB applied on the back	3825	99	2.60	[32]
	1	Aq.	48	48, 72	Finn Chambers on Sanpor applied on the upper back	373	11	2.90	[33]
	1	Aq.	48	48, 72, 96, 168	n/a	1471	43	2.90	[25]
	1	Aq.	48	48, 96	Aluminium chambers carried out on the upper back	33	1	3.00	[45]
	1	Aq.	48	48, 72, 96, 168	Finn Chambers (8mm) on Scanpor tape	18 179	555	3.10	[38]
	1	Aq.	48	96	Finn Chambers applied to the back of the patients	618	20	3.20	[44]
	1	Aq.	48	48, 96	Finn Chambers on Scanpor tape applied on back	125	4	3.20	[23]
	1	Aq.	48	48, 96	Finn Chambers on Scanpor applied to the upper back	91	3	3.30	[43]
	1	Aq.	48	48, 72, 96, 168	n/a	1468	61	4.20	[25]
	1	Aq.	48	48, 96	Finn Chambers on Scanpor tape to the upper back	6845	315	4.60	[29]
	1	Aq.	48	48, 96	IQ Chambers	327	16	4.90	[34]

	1	Pet.	48	120, 168	IQ Ultra Chambers applied on the patient's back	1608	84	5.20	[46]
	1	Aq.	48	48, 72	Finn Chambers applied on the upper back	2295	131	5.70	[20]
	1	Aq.	48	48, 96	n/a	385	23	6.00	[16]
	1	Aq.	48	48, 96	Finn Chambers on Scanpor tape applied to the areas of the back	342	22	6.40	[42]
	1	Aq.	48	48, 72	Aluminium patch test chambers mounted on Micropore carried out on the upper back	200	13	6.50	[26]
	1	Aq.	48	48, 72, 96, 168	Finn Chambers (8 mm), applied on the upper aspect of the back	3093	241	7.80	[21]
	1	Aq.	48	48, 72, 96, 168	Finn Chambers (8 mm), applied on the upper back with Scanpor tape	1321	104	7.90	[19]
	1	Aq.	48	48, 72, 96, 168	Finn Chambers (8 mm), applied on the upper back with Scanpor tape	4909	412	8.40	[19]
	1	Aq.	48	48, 72, 96, 168	Finn Chambers (8 mm), applied on the upper aspect of the back	4445	400	9.00	[21]

	1	Aq.	48	48, 72, 96, 168	Finn Chambers (8 mm), applied on the upper aspect of the back	3836	345	9.00	[21]
**Fragrance Mix I**	8	Pet.	48	48, 96	n/a	50	1	2.00	[16]
	8	Pet.	48	n/a	TRUE Test and Finn Chambers	3119	82	2.60	[5]
	7	Pet.	48	48, 96	Finn Chambers on Scanpor tape applied on back	125	5	4.00	[23]
	8	Pet.	48	48, 96, 168	Finn Chambers fixed with Scanpor	1253	56	4.50	[31]
	8	Pet.	48	48, 96	n/a	385	19	4.90	[16]
	8	Pet.	48	72, 144, 168	IQ Chambers from Chemotechnique Diagnostics AB applied on the back	3112	162	5.20	[32]
	8	Pet.	48	48, 72, 96, 168	n/a	1991	128	6.40	[24]
	8	Pet.	48	48, 72, 96	Finn Chambers (8 mm) on Scanpor tape	1699	111	6.50	[48]
	8	Pet.	48	48, 72, 96, 168	n/a	1467	97	6.60	[25]
	8	Pet.	48	72, 168	Finn Chambers on Scanpor tape	2598	174	6.70	[47]
	8	Pet.	48	72, 96	n/a	1896	150	6.90	[30]
	8	Pet.	48	48, 72	Finn Chambers on Scanpor tape applied to upper back	2758	190	6.90	[27]
	8	Pet.	48	120, 168	IQ Ultra Chambers applied on the patient's back	1608	112	7.00	[46]

	8	Pet.	48	72, 96	n/a	36 961	2694	7.29	[12]
	8	Pet.	48	48, 72	Aluminium patch test chambers mounted on Micropore carried out on the upper back	200	15	7.50	[26]
	8	Pet.	48	48, 72	Finn Chambers on Sanpor applied on the upper back	373	30	8.00	[33]
	8	Pet.	48	72, 96	n/a	700	57	8.10	[30]
	8	Pet.	48	72, 96	n/a	10 124	859	8.40	[10]
	8	Pet.	48	48, 72, 96, 168	n/a	1399	117	8.40	[35]
	8	Pet.	48	96	Finn Chambers applied to the back of the patients	618	54	8.70	[44]
	8	Pet.	48	72, 96	n/a	972	91	9.40	[18]
	8	Pet.	48	72, 96	n/a	2248	211	9.40	[37]
	8	Pet.	48	48, 72, 96	Van der Bend patch test chambers applied on the back with Micropore and fixed with Fixomull and Mefix	13 114	1259	9.60	[8]
	8	Pet.	48	48	Finn Chambers on Scanpor tape	422	41	9.70	[1]
	8	Pet.	48	72, 96	n/a	2007	205	10.00	[37]
	8	Pet.	48	48, 72, 96, 168	Finn Chambers (8 mm), applied on the upper back with Scanpor tape	1323	138	10.40	[19]

	8	Pet.	48	48, 72, 96, 168	Finn Chambers (8 mm), applied on the upper aspect of the back	3092	322	10.40	[21]
	8	Pet.	48	48, 72, 96, 168	Finn Chambers (8 mm), applied on the upper back with Scanpor tape	4896	509	10.40	[19]
	8	Pet.	48	72, 96	n/a	700	57	11.10	[30]
	8	Pet.	48	48, 96	IQ Chambers	327	37	11.30	[34]
	8	Pet.	48	48, 72, 96, 168	Finn Chambers (8 mm), applied on the upper aspect of the back	940	106	11.30	[4]
	8	Pet.	48	48, 72, 96, 168	Finn Chambers (8 mm), applied on the upper back with Scanpor tape	3844	434	11.30	[19]
	8	Pet.	48	48, 72, 96, 168	Finn Chambers (8 mm), applied on the upper aspect of the back	4439	510	11.50	[21]
	8	Pet.	48	72, 144, 168	IQ Chambers from Chemotechnique Diagnostics AB applied on the back	3112	548	17.60	[32]
	8	Pet.	48	48, 96	Finn Chambers applied on the back	312	57	18.30	[3]
	8	Pet.	48	72, 144, 168	IQ Chambers from Chemotechnique Diagnostics AB applied on the back	3825	757	19.80	[32]

	8	Pet.	48	72	Finn Chambers and TRUE Test applied on the back	3662	784	21.40	[32]
	8	Pet.	48	72, 120	Finn Chambers on Scanpor	41	11	27.00	[21]
	8	Pet.	48	48, 96	Aluminium chambers carried out on the upper back	33	9	27.20	[45]
	8	Pet.	48	48, 120	Finn Chambers (8 mm), applied on the upper back with Scanpor tape	167	79	47.30	[13]
	8	Pet.	48	48, 120	n/a	204	154	75.50	[44]
	8	Pet.	48	48, 120	Finn Chambers secured to the skin with Scanpor tape applied to the upper back	178	140	78.70	[7]
	8	Pet.	48	48, 96	Finn Chambers on Scanpor applied to the upper back	91	85	93.00	[43]
**Fragrance Mix II**	14	Pet.	48	72, 168	Finn Chambers on Scanpor tape	2598	21	0.80	[47]
	2.8	Pet.	48	48, 72, 96	Finn Chambers (8 mm) on Scanpor tape	1701	22	1.30	[48]
	14	Pet.	48	48, 96, 168	Finn Chambers fixed with Scanpor	1253	7	1.50	[31]
	14	Pet.	48	n/a	TRUE Test and Finn Chambers	3119	60	1.90	[5]
	14	Pet.	48	72, 96,168	Finn Chambers (8 mm) on Scanpor tape	10 010	189	1.90	[49]

	14	Pet.	48	120, 168	IQ Ultra Chambers applied on the patient’s back	1608	48	2.60	[46]
	14	Pet.	48	48, 72, 96	Finn Chambers (8 mm) on Scanpor tape	1701	50	2.90	[48]
	14	Pet.	48	72, 96	n/a	14 728	560	3.80	[10]
	14	Pet.	48	48, 72, 96, 168	n/a	1231	49	4.00	[25]
	28	Pet.	48	48, 72, 96	Finn Chambers (8 mm) on Scanpor tape	1701	70	4.10	[48]
	14	Pet.	48	48, 72, 96, 168	n/a	1233	54	4.40	[25]
	14	Pet.	48	72, 96	n/a	1895	86	4.40	[30]
	14	Pet.	48	72, 96	n/a	35 738	1748	4.89	[12]
	14	Pet.	24, 48	72, 96	n/a	35 633	1742	4.90	[50]
	14	Pet.	48	48, 72, 96, 168	Finn Chambers (8 mm), applied on the upper aspect of the back	192	10	5.20	[4]
	14	Pet.	48	72, 96	n/a	10 124	559	5.50	[10]
	14	Pet.	48	72, 120	Finn Chambers on Scanpor	17	1	5.90	[21]
	14	Pet.	48	48, 72, 96	Van der Bend patch test chambers applied on the back with Micropore and fixed with Fixomull and Mefix	3416	205	6.00	[8]
	14	Pet.	48	48, 72, 96, 168	Finn Chambers (8 mm), applied on the upper aspect of the back	2713	165	6.10	[21]

	14	Pet.	48	72, 96	n/a	2248	148	6.60	[37]
	14	Pet.	48	48, 72, 96, 168	n/a	1467	97	6.60	[25]
	14	Pet.	48	72, 96	n/a	698	33	7.00	[30]
	14	Pet.	48	72, 96	n/a	1625	121	7.40	[37]
	14	Pet.	48	48, 72, 96	Finn Chambers (8 mm) on Scanpor tape	1701	128	7.50	[48]
	14	Pet.	48	48, 72, 96, 168	n/a	1470	115	7.80	[25]
	14	Pet.	48	48, 96	Finn Chambers applied on the back	312	25	8.00	[3]
**Geraniol**	2	Pet.	48	n/a	TRUE Test and Finn Chambers	3119	13	0.40	[5]
	1	Pet.	48	72	n/a	2063	10	0.50	[11]
	2	Pet.	48	48, 96	Finn Chambers applied on the back	312	8	2.60	[3]
	5	Pet.	48	48, 120	Finn Chambers (8 mm), applied on the upper back with Scanpor tape	167	5	3.00	[13]
	2	Pet.	48	48, 72, 96	Van der Bend patch test chambers applied on the back with Micropore and fixed with Fixomull and Mefix	940	52	5.50	[8]
	1	Pet.	48	72, 96	n/a	405	30	7.40	[10]
	2	Pet.	48	n/a	n/a	59	11	18.60	[9]
	2	Pet.	48	48, 96, 168	Finn Chambers fixed with Scanpor	86	17	19.70	[31]
**Hexyl cinnamal**	10	Pet.	48	n/a	TRUE Test and Finn Chambers	3119	8	0.30	[5]

	10	Pet.	48	48, 72, 96, 168	Finn Chambers (8 mm), applied on the back with Scanpor tape	1503	9	0.60	[2]
	10	Pet.	48	48, 96	Finn Chambers applied on Scanpor tape to the upper back of patients	308	2	0.60	[6]
	10	Pet.	48	72, 96	n/a	137	10	7.30	[10]
	10	Pet.	48	48, 72, 96	Van der Bend patch test chambers applied on the back with Micropore and fixed with Fixomull and Mefix	205	20	9.70	[8]
**Hydroxycitronellal**	2	Pet.	48	n/a	TRUE Test and Finn Chambers	3119	15	0.50	[5]
	1	Pet.	48	48, 72, 96, 168	Finn Chambers (8 mm), applied on the back with Scanpor tape	1498	9	0.60	[2]
	1	Pet.	24, 48	72	n/a	2063	27	1.30	[11]
	1	Pet.	48	48, 96	Finn Chambers on Scanpor applied to the upper back	40	1	2.50	[43]
	2	Pet.	48	48, 72, 96	Van der Bend patch test chambers applied on the back with Micropore and fixed with Fixomull and Mefix	940	24	2.60	[8]

	1	Pet.	48	48, 96	Finn Chambers applied on the back	312	12	3.80	[3]
	1	Pet.	48	72, 96	n/a	607	26	4.30	[37]
	7	Pet.	48	48, 120	n/a	216	13	6.00	[44]
	5	Pet.	48	48, 96, 168	Finn Chambers fixed with Scanpor	86	6	6.90	[31]
	4	Pet.	48	48, 120	Finn Chambers (8 mm), applied on the upper back with Scanpor tape	167	23	13.80	[13]
	1	Pet.	48	72, 96	n/a	405	59	14.60	[10]
	2	Pet.	48	n/a	n/a	59	23	40.00	[9]
**Imidazolidynyl urea**	2	Pet.	48	48, 96	Komory IQ Chambers Chemotechnique Diagnostics	405	1	0.20	[14]
	2	Pet.	48	48, 96	Finn Chambers applied on Scanpor tape to the upper back of patients	308	1	0.30	[6]
	2	Pet.	48	48, 96	Finn Chambers secured with Scanpor tape and left on the back	3062	15	0.50	[38]
	2	Pet.	24, 48	72	Finn Chambers (8 mm) on Scan-por (19 departments), Leukotest, Hal, Curatest, Haye, Intradex Service BV, Alphen aan den Rijn,	11 452	64	0.60	[17]
	2	Pet.	48	48, 72, 96, 168	n/a	1398	8	0.60	[35]

	2	Pet.	48	72, 96	n/a	78 670	479	0.60	[28]
	2	Pet.	48	72, 96	Curatest Lohman&Rauscher, Rengsdorf Germany	1927	13	0.70	[15]
	1	Pet.	48	48, 96	Finn Chambers apllied with Scanpor tape to the upper back	2298	16	0.70	[40]
	2	Pet.	48	72, 96	n/a	6981	55	0.70	[10]
	2	Pet.	48	48, 72, 96, 168	Finn Chambers (8mm) on Scanpor tape	11 271	93	0.80	[38]
	2	Pet.	48	48, 96	Finn Chambers (8 mm) or IQ Chambers	9360	84	0.90	[41]
	2	Pet.	48	48, 96	n/a	385	4	1.00	[16]
	2	Pet.	48	48, 72	Finn Chambers applied on the upper back	2295	23	1.00	[20]
	2	Pet.	48	48, 72	Finn Chambers on Scanpor tape applied to upper back	2758	27	1.00	[27]
	2	Aq.	48	48, 96	Finn Chambers fixed with Scanpor tape	3900	41	1.05	[35]
	2	Aq.	48	48, 72, 96, 168	Finn Chambers (8 mm), applied on the upper aspect of the back	4447	58	1.30	[21]
	2	Aq.	48	48, 72, 96, 168	Finn Chambers (8 mm), applied on the upper back with Scanpor tape	1322	22	1.70	[19]

	2	Aq.	48	48, 72, 96, 168	Finn Chambers (8 mm), applied on the upper aspect of the back	3069	52	1.70	[21]
	2	Aq.	48	48, 72, 96, 168	Finn Chambers on Scanpor tape applied to a patient’s upper back	943	17	1.80	[4]
	2	Aq.	48	48, 72, 96, 168	Finn Chambers (8 mm), applied on the upper back with Scanpor tape	4909	88	1.80	[19]
	2	Pet.	48	48, 96	Finn Chambers on Scanpor tape to the upper back	6845	130	1.90	[29]
	2	Pet.	48	48, 96	IQ Chambers	327	7	2.10	[34]
	2	Aq.	48	48, 72, 96, 168	Finn Chambers (8 mm), applied on the upper aspect of the back	3843	81	2.10	[21]
	2	Pet.	48	48, 72, 96, 168	Finn Chambers on Scanpor tape applied to a patient’s upper back	941	22	2.30	[4]
	2	Pet.	48	48, 72, 96, 168	Finn Chambers (8 mm), applied on the upper aspect of the back	3091	77	2.50	[21]
	2	Pet.	48	48, 72, 96, 168	Finn Chambers (8 mm), applied on the upper aspect of the back	3819	106	2.80	[21]

	2	Pet.	48	48, 96	Finn Chambers (8 mm), applied on the upper back with Scanpor tape	584	17	2.90	[22]
	2	Pet.	48	48, 72, 96, 168	Finn Chambers (8 mm), applied on the upper aspect of the back	4438	129	2.90	[21]
	2	Pet.	48	48, 72, 96, 168	Finn Chambers (8 mm), applied on the upper back with Scanpor tape	4897	147	3.00	[19]
	2	Pet.	48	48, 72, 96, 168	Finn Chambers (8 mm), applied on the upper back with Scanpor tape	1321	44	3.30	[19]
	2	Pet.	48	48, 96	Finn Chambers on Scanpor tape applied to the areas of the back	342	13	3.80	[42]
**Iodopropynyl butylcarbamate**	0.5	Pet.	48	48, 72, 96, 168	Finn Chambers (8mm) on Scanpor tape	10 085	41	0.40	[38]
	0.1	Pet.	48	48, 96	Finn Chambers (8 mm), applied on the upper back with Scanpor tape	584	3	0.50	[22]
	0.2	Pet.	48	48, 72, 96, 168	n/a	1451	8	0.60	[25]
	0.2	Pet.	48	48, 96	Komory IQ Chambers Chemotechnique Diagnostics	405	3	0.70	[14]

	0.2	Pet.	48	48, 72, 96, 168	n/a	1390	10	0.70	[35]
	0.1	Pet.	48	48, 72, 96, 168	Finn Chambers on Scanpor tape applied to a patient's upper back	871	6	0.70	[4]
	0.2	Pet.	48	48, 72, 96, 168	Finn Chambers (8 mm), applied on the upper aspect of the back	1229	12	0.98	[21]
	0.2	Pet.	48	72, 96	n/a	6974	77	1.10	[10]
	0.2	Pet.	48	48, 72, 96, 168	n/a	1452	17	1.20	[25]
	0.2	Pet.	48	72, 96	n/a	1697	32	1.90	[37]
	0.5	Pet.	48	48, 72, 96, 168	Finn Chambers (8 mm), applied on the upper aspect of the back	4435	106	2.40	[21]
	0.2	Pet.	48	48, 72, 96, 168	Finn Chambers (8 mm), applied on the upper aspect of the back	118	4	3.40	[21]
**Isoeugenol**	2	Pet.	48	n/a	TRUE Test and Finn Chambers	3119	21	0.70	[5]
	1	Pet.	48	48, 72, 96, 168	Finn Chambers (8 mm), applied on the back with Scanpor tape	1502	14	0.90	[2]
	1	Pet.	24, 48	72	n/a	2063	26	1.30	[11]
	1	Pet.	48	48, 96	Finn Chambers applied on the back	312	7	2.20	[3]
	1	Pet.	48	72, 96	n/a	621	32	5.20	[37]

	2	Pet.	48	48, 96, 168	Finn Chambers fixed with Scanpor	86	11	12.80	[31]
	4	Pet.	48	48, 120	Finn Chambers (8 mm), applied on the upper back with Scanpor tape	167	23	13.80	[13]
	2	Pet.	48	48, 72, 96	Van der Bend patch test chambers applied on the back with Micropore and fixed with Fixomull and Mefix	940	160	17.00	[8]
	1	Pet.	48	72, 96	n/a	405	78	19.30	[10]
	1	Pet.	48	48, 96	Finn Chambers on Scanpor applied to the upper back	40	8	20.00	[43]
	2	Pet.	48	n/a	n/a	59	21	35.60	[9]
**Isopropyl myristate**	20	Pet.	48	48, 72, 96, 168	Finn Chambers (8 mm), applied on the upper back with Scanpor tape	3844	8	0.20	[19]
	20	Pet.	48	48, 72, 96, 168	Finn Chambers (8 mm), applied on the upper aspect of the back	3086	6	0.20	[21]
	20	Pet.	48	48, 72, 96, 168	Finn Chambers (8 mm), applied on the upper aspect of the back	944	4	0.40	[4]
**Lanolin**	30	Pet.	48	48, 72, 96, 168	n/a	1399	12	0.90	[35]
	30	Pet.	48	72, 168	Finn Chambers on Scanpor tape	2598	18	0.70	[47]

	30	Pet.	48	48, 72, 96, 168	n/a	1470	11	0.70	[25]
	30	Pet.	48	96	Finn Chambers applied to the back of the patients	618	16	2.60	[44]
	30	Pet.	48	72, 96	n/a	1900	33	1.70	[30]
	30	Pet.	48	72, 96	n/a	14 728	265	1.80	[10]
	30	Pet.	48	48, 72, 96, 168	Finn Chambers (8 mm), applied on the upper aspect of the back	935	26	2.80	[4]
	30	Pet.	48	72, 120	Finn Chambers on Scanpor	34	1	2.90	[21]
	30	Pet.	48	48, 96	Finn Chambers on Scanpor tape applied on back	125	0	0.00	[23]
	30	Pet.	48	48, 72, 96, 168	n/a	1469	7	0.50	[25]
	30	Pet.	48	72, 96	n/a	708	10	1.40	[30]
	30	Pet.	48	120, 168	IQ Ultra Chambers applied on the patient's back	1608	24	1.50	[46]
	30	Pet.	48	72, 96	n/a	2058	44	2.10	[37]
	30	Pet.	48	72, 96	n/a	10 124	246	2.40	[10]
**Limonene**	2	Pet.	24, 48	72	n/a	2396	3	0.10	[11]
	2	Pet.	48	72, 96	n/a	1241	4	0.30	[12]
	2	Pet.	48	48, 96	Finn Chambers applied on Scanpor tape to the upper back of patients	308	2	0.60	[6]
	2	Pet.	48	48, 96	Finn Chambers applied on the back	312	2	0.60	[3]

**Linalool**	10	Pet.	48	48, 72, 96, 168	Finn Chambers (8 mm), applied on the back with Scanpor tape	1397	1	0.10	[2]
	10	Pet.	48	72, 96	n/a	985	2	0.20	[12]
	10	Pet.	24, 48	72	n/a	2401	7	0.30	[11]
	10	Pet.	48	48, 96	Finn Chambers applied on Scanpor tape to the upper back of patients	308	2	0.60	[6]
**Hydroxyisohexyl 3-cyclohexene carboxaldehyde (Lyral)**	5	Pet.	48	72, 96,168	Finn Chambers (8 mm) on Scanpor tape	10 010	23	0.20	[49]
	5	Pet.	48	72, 168	Finn Chambers on Scanpor tape	2598	13	0.50	[47]
	2	Pet.	48	48, 72, 96, 168	Finn Chambers (8 mm), applied on the upper aspect of the back	444	5	1.10	[4]
	5	Pet.	48	48, 72, 96, 168	Finn Chambers (8 mm), applied on the back with Scanpor tape	1502	24	1.60	[2]
	5	Pet.	48	48	Finn Chambers on Scanpor tape	422	7	1.70	[1]
	5	Pet.	48	72, 96	n/a	14 728	265	1.80	[10]
	5	Pet.	48	72, 96	n/a	704	13	1.80	[30]
	5	Pet.	48	48, 72, 96	Van der Bend patch test chambers applied on the back with Micropore and fixed with Fixomull and Mefix	3927	82	2.10	[8]

	5	Pet.	48	48, 72, 96, 168	n/a	1457	34	2.30	[25]
	5	Pet.	48	48, 72, 96, 168	n/a	1461	33	2.30	[25]
	5	Pet.	48	48, 72, 96, 168	Finn Chambers (8 mm), applied on the upper aspect of the back	354	8	2.30	[4]
	5	Pet.	48	72, 96	n/a	10 124	242	2.40	[10]
	5	Pet.	24, 48	72	n/a	21 325	502	2.40	[11]
	5	Pet.	48	120, 168	IQ Ultra Chambers applied on the patient's back	1608	38	2.40	[46]
	5	Pet.	48	72, 96	n/a	37 271	898	2.40	[12]
	5	Pet.	48	48, 72, 96, 168	n/a	1913	56	2.80	[24]
	5	Pet.	48	72, 96	n/a	1905	5	2.80	[30]
	5	Pet.	48	48, 96	Finn Chambers applied on the back	312	10	3.20	[3]
	5	Pet.	48	48, 72, 96, 168	n/a	1392	51	3.70	[35]
	5	Pet.	48	72, 96	n/a	2043	93	4.60	[37]
	5	Pet.	48	48, 96, 168	Finn Chambers fixed with Scanpor	86	7	8.10	[31]
	5	Pet.	48	48, 72, 96	Van der Bend patch test chambers applied on the back with Micropore and fixed with Fixomull and Mefix	205	58	28.30	[8]
**m-Aminophenol**	1	Pet.	48	48, 72, 168	Finn Chambers on Scanpor tape	198	5	2.50	[51]

**Methylchloroisothiazolinone**	0.01	Aq.	48	48, 96	IQ Chambers	327	13	4.00	[34]
**Methylchloroisothiazolinone/ Methylisothiazolinone**	0.01	Aq.	48	48, 96	Finn Chambers applied on Scanpor tape to the upper back of patients	308	1	0.30	[6]
	0.5	Aq.	48	48, 96	Finn Chambers on Scanpor tape	125	1	0.80	[23]
	0.67	Aq.	48	48, 72	Aluminium patch test chambers mounted on Micropore carried out on the upper back	200	2	1.00	[26]
	0.01	Aq.	48	48, 72, 96, 168	n/a	1993	24	1.20	[24]
	0.01	Aq.	48	72, 96	Curatest Lohman&Rauscher, Rengsdorf Germany	1927	26	1.40	[15]
	0.01	Aq.	48	48, 72, 96, 168	n/a	1471	24	1.60	[25]
	0.01	Aq.	48	48, 96	Finn Chambers (8 mm) or IQ Chambers	9345	155	1.70	[41]
	0.01	Aq.	48	72, 168	Finn Chambers on Scanpor tape	2598	47	1.80	[47]
	0.01	Aq.	48	48, 72, 96, 168	Finn Chambers (8mm) on Scanpor tape	18 058	323	1.80	[38]
	0.01	Aq.	48	72, 96	n/a	14 728	295	2.00	[10]
	0.02	Aq.	48	72, 144, 168	IQ Chambers from Chemotechnique Diagnostics AB applied on the back	3112	62	2.00	[32]

	0.01	Aq.	48	72, 96	n/a	1906	43	2.10	[30]
	0.01	Aq.	48	48, 72, 96, 168	Finn Chambers (8 mm), applied on the upper back with Scanpor tape	4895	113	2.30	[19]
	0.01	Aq.	48	48, 96	Finn Chambers on Scanpor tape to the upper back	5927	136	2.30	[29]
	0.01	Aq.	48	72, 96	n/a	1906	43	2.30	[30]
	0.01	Aq.	48	72, 96	n/a	121 136	2805	2.32	[28]
	0.01	Aq.	48	48, 96	Finn Chambers secured with Scanpor tape and left on the back	3062	73	2.40	[38]
	0.01	Aq.	48	96	Finn Chambers applied to the back of the patients	618	15	2.43	[44]
	0.02	Aq.	48	72, 144, 168	IQ Chambers from Chemotechnique Diagnostics AB applied on the back	3825	92	2.40	[32]
	0.01	Aq.	24, 48	72	Finn Chambers (8 mm) on Scan-por (19 departments), Leukotest, Hal, Curatest, Haye, Intradex Service BV, Alphen aan den Rijn,	27 331	717	2.60	[17]
	0.01	Aq.	48	48, 72, 96, 168	n/a	1399	39	2.80	[35]
	0.01	Aq.	48	48, 72, 96, 168	Finn Chambers (8 mm), applied on the	4437	124	2.80	[21]

					upper aspect of the back				
	1.34	Aq.	48	48, 96	Aluminium chambers carried out on the upper back	33	1	3.00	[45]
	0.01	Aq.	48	48, 72, 96, 168	Finn Chambers (8 mm), applied on the upper aspect of the back	3740	112	3.00	[21]
	0.01	Aq.	48	48, 72, 96, 168	Finn Chambers (8 mm), applied on the upper back with Scanpor tape	1312	41	3.10	[19]
	0.02	Aq.	48	72	Finn Chambers and TRUE Test applied on the back	3662	114	3.10	[32]
	0.01	Aq.	48	72, 96	n/a	10 124	304	3.20	[10]
	0.01	Aq.	48	48, 72, 96, 168	Finn Chambers (8 mm), applied on the upper aspect of the back	3091	105	3.40	[21]
	0.01	Aq.	48	72, 96	n/a	6797	232	3.40	[10]
	0.01	Aq.	48	48, 96	Komory IQ Chambers Chemotechnique Diagnostics	405	14	3.50	[14]
	0.01	Aq.	48	48, 96	IQ Chambers	327	13	4.00	[34]
	0.01	Aq.	48	48, 72, 96, 168	n/a	1469	61	4.20	[25]
	0.01	Aq.	48	48, 72	Finn Chambers on Sanpor applied on the upper back	208	9	4.30	[33]

	0.01	Aq.	48	48, 72, 96, 168	Finn Chambers on Scanpor tape applied to a patient's upper back	935	40	4.30	[4]
	0.01	Aq.	48	48, 96	Finn Chambers on Scanpor applied to the upper back	91	4	4.40	[43]
	0.01	Aq.	48	72, 96	n/a	2248	99	4.40	[37]
	0.01	Aq.	48	72, 96	n/a	2048	103	5.00	[37]
	0.02	Aq.	48	48, 96	Finn Chambers (8 mm), applied on the upper back with Scanpor tape	584	32	5.50	[22]
	0.01	Pet.	48	120, 168	IQ Ultra Chambers applied on the patient's back	1608	88	5.50	[46]
	0.67	Aq.	48	48, 72	Finn Chambers applied on the upper back	2295	126	5.50	[20]
	0.01	Aq.	48	72, 96	n/a	706	39	5.52	[30]
	0.01	Aq.	48	48, 96	n/a	50	3	5.90	[16]
	0.01	Aq.	48	48, 96	n/a	385	23	6.00	[16]
**Methyldibromo Glutaronitrile (MDBGN)**	0.30	Pet.	48	72, 168	Finn Chambers on Scanpor tape	2598	18	0.70	[47]
	0.30	Pet.	48	48, 96	Finn Chambers (8 mm) or IQ Chambers	8496	93	1.10	[41]
	0.30	Pet.	48	48, 96	Komory IQ Chambers Chemotechnique Diagnostics	405	7	1.70	[14]
	0.30	Pet.	48	72, 96	n/a	1374	24	1.80	[30]
	0.30	Pet.	48	48, 72, 96, 168	n/a	1858	34	1.80	[24]

	0.30	Pet.	48	72, 96	n/a	525	14	2.70	[30]
	0.30	Pet.	48	72, 96	n/a	14 728	412	2.80	[10]
	0.30	Pet.	48	48, 72, 96, 168	Finn Chambers (8mm) on Scanpor tape	7511	276	3.70	[38]
	0.30	Pet.	48	48, 72, 96, 168	n/a	1398	61	4.40	[35]
	0.30	Pet.	48	48, 72, 96, 168	n/a	1469	65	4.40	[25]
	0.30	Pet.	48	72, 96	n/a	1680	79	4.70	[37]
	0.30	Pet.	48	72, 96	n/a	53 545	2695	5.00	[28]
	0.30	Pet.	48	48, 72, 96, 168	n/a	1466	89	6.10	[25]
**Methylisothiazolinone**	0.05	Aq.	48	72, 96	n/a	13 433	215	1.60	[28]
	0.01	Aq.	48	72, 96	n/a	933	18	1.90	Britt
	0.05	Aq.	48	48, 72, 96, 168	n/a	1060	25	2.40	[35]
	0.05	Aq.	48	72, 96	n/a	4089	157	3.80	[10]
	0.05	Aq.	48	48, 72, 96, 168	n/a	919	37	4.00	[35]
	0.05	Aq.	48	72, 96	n/a	1025	44	4.29	[37]
	0.02	Aq.	48	48, 96	Komory IQ Chambers Chemotechnique Diagnostics	405	19	4.70	[14]
	0.02	Aq.	48	120, 168	IQ Ultra Chambers applied on the patient's back	1608	112	7.00	[46]
**Mercaptobenzothiazole**	2	Pet.	48	72, 96	n/a	1904	3	0.20	[30]
	2	Pet.	48	72, 168	Finn Chambers on Scanpor tape	2598	8	0.30	[47]

	2	Pet.	48	72, 144, 168	IQ Chambers from Chemotechnique Diagnostics AB applied on the back	3825	15	0.40	[32]
	2	Pet.	48	72, 144, 168	IQ Chambers from Chemotechnique Diagnostics AB applied on the back	3112	13	0.40	[32]
	2	Pet.	48	48, 72, 96, 168	n/a	1398	9	0.60	[35]
	2	Pet.	48	72	Finn Chambers and TRUE Test applied on the back	3662	22	0.60	[32]
	2	Pet.	48	48, 72, 96, 168	n/a	1993	12	0.60	[24]
	2	Pet.	48	72, 96	n/a	703	4	0.60	[30]
	2	Pet.	48	48, 72	Finn Chambers on Sanpor applied on the upper back	337	3	0.90	[33]
	2	Pet.	48	48, 72, 96, 168	n/a	1471	14	1.00	[25]
	2	Pet.	48	48, 72, 96, 168	n/a	1468	16	1.10	[25]
	2	Pet.	48	72, 120	Finn Chambers on Scanpor	34	1	2.90	[21]
	2	Pet.	48	96	Finn Chambers applied to the back of the patients	618	20	3.20	[44]
**Mercapto mix**	2	Pet.	48	72, 168	Finn Chambers on Scanpor tape	2598	5	0.20	[47]
	2	Pet.	48	48, 96	IQ Chambers	327	1	0.30	[34]
	2	Pet.	48	48, 72, 96, 168	Finn Chambers (8 mm), applied on the	3091	15	0.50	[21]

					upper aspect of the back				
	2	Pet.	48	48, 72, 96, 168	Finn Chambers (8 mm), applied on the upper back with Scanpor tape	3842	23	0.60	[19]
	2	Pet.	48	48, 96	Finn Chambers on Scanpor tape applied on back	125	1	0.80	[23]
	2	Pet.	48	48, 72, 96, 168	n/a	1471	13	0.90	[25]
	2	Pet.	48	48, 72, 96, 168	n/a	1398	14	1.00	[35]
	2	Pet.	48	48, 72	Finn Chambers on Sanpor applied on the upper back	373	6	1.60	[33]
	2	Pet.	48	48, 72	Aluminium patch test chambers mounted on Micropore carried out on the upper back	200	10	5.00	[26]
**Myroxylon pereirae resin**	25	Pet.	48	n/a	TRUE Test and Finn Chambers	3119	23	0.70	[5]
	25	Pet.	48	48, 72	Aluminium patch test chambers mounted on Micropore carried out on the upper back	200	3	1.50	[26]
	25	Pet.	48	48, 96	Finn Chambers on Scanpor tape applied on back	125	2	1.60	[23]

	25	Pet.	48	48, 72, 96, 168	n/a	1469	30	2.00	[25]
	25	Pet.	48	48, 72, 96, 168	n/a	1471	35	2.40	[25]
	25	Pet.	48	48, 72, 96, 168	n/a	1993	60	3.00	[24]
	25	Pet.	48	48, 72 96, 168	n/a	1397	42	3.00	[35]
	25	Pet.	48	48, 72 96, 168	n/a	1400	43	3.10	[35]
	25	Pet.	48	48, 96	n/a	50	2	4.00	[16]
	25	Pet.	48	48, 72	Finn Chambers on Sanpor applied on the upper back	373	15	4.00	[33]
	25	Pet.	48	72, 144, 168	IQ Chambers from Chemotechnique Diagnostics AB applied on the back	3112	149	4.80	[32]
	25	Pet.	48	72	Finn Chambers and TRUE Test applied on the back	3662	179	4.90	[32]
	25	Pet.	48	72, 96	n/a	14 728	795	5.40	[10]
	25	Pet.	48	48, 96	n/a	385	21	5.50	[16]
	25	Pet.	48	120, 168	IQ Ultra Chambers applied on the patient’s back	1608	90	5.60	[46]
	25	Pet.	48	72, 96	n/a	1898	131	6.10	[30]
	25	Pet.	48	48, 96, 168	Finn Chambers fixed with Scanpor	1253	80	6.40	[31]
	25	Pet.	48	72, 96	n/a	972	63	6.50	[18]

	25	Pet.	48	72, 144, 168	IQ Chambers from Chemotechnique Diagnostics AB applied on the back	3825	249	6.50	[32]
	25	Pet.	48	48, 96	Finn Chambers secured with Scanpor tape and left on the back	3062	205	6.70	[38]
	25	Pet.	48	48, 96	Finn Chambers applied on the back	312	21	6.80	[3]
	25	Pet.	48	72, 96	n/a	705	37	6.90	[30]
	25	Pet.	48	72, 96	n/a	1898	131	6.90	[30]
	25	Pet.	48	72, 96	n/a	2248	157	7.00	[37]
	25	Pet.	48	72, 96	n/a	2048	145	7.10	[37]
	25	Pet.	48	48	Finn Chambers on Scanpor tape	422	31	7.30	[1]
	25	Pet.	48	72, 96	n/a	10 124	755	7.40	[10]
	25	Pet.	48	72, 96	n/a	36 919	2950	8.00	[12]
	25	Pet.	48	48, 72, 96, 168	Finn Chambers (8 mm), applied on the upper back with Scanpor tape	1322	149	11.30	[19]
	25	Pet.	48	48, 72, 96, 168	Finn Chambers (8 mm), applied on the upper back with Scanpor tape	4910	569	11.60	[19]
	25	Pet.	48	48, 72, 96, 168	Finn Chambers (8 mm), applied on the upper aspect of the back	4449	529	11.90	[21]
	25	Pet.	48	48, 72, 96, 168	Finn Chambers (8 mm), applied on the	936	116	12.40	[4]

					upper aspect of the back				
	25	Pet.	48	48, 72, 96, 168	Finn Chambers (8 mm), applied on the upper aspect of the back	3082	388	12.60	[21]
	25	Pet.	48	48, 72, 96, 168	Finn Chambers (8 mm), applied on the upper aspect of the back	3837	526	13.70	[21]
	25	Pet.	48	48, 96	Finn Chambers on Scanpor applied to the upper back	91	19	20.90	[43]
	25	Pet.	48	72, 120	Finn Chambers on Scanpor	41	10	24.00	[21]
	25	Pet.	48	48, 120	Finn Chambers (8 mm), applied on the upper back with Scanpor tape	125	34	27.20	[13]
**Neomycin sulfate**	20	Pet.	48	48, 72	Finn Chambers on Scanpor tape applied to upper back	2758	17	0.60	[27]
	20	Pet.	48	72, 120	Finn Chambers on Scanpor	34	4	11.80	[21]
	20	Pet.	48	48, 72, 96, 168	Finn Chambers (8 mm), applied on the upper back with Scanpor tape	3834	452	11.80	[19]
	20	Pet.	48	72	Finn Chambers and TRUE Test applied on the back	3662	70	1.90	[32]

	20	Pet.	48	48, 96	Finn Chambers on Scanpor applied to the upper back	91	9	9.90	[43]
	20	Pet.	48	72, 144, 168	IQ Chambers from Chemotechnique Diagnostics AB applied on the back	3112	12	0.40	[32]
	20	Pet.	48	72, 168	Finn Chambers on Scanpor tape	2598	13	0.50	[47]
	20	Pet.	48	72, 144, 168	IQ Chambers from Chemotechnique Diagnostics AB applied on the back	3825	46	1.20	[32]
	20	Pet.	48	48, 72, 96, 168	n/a	1468	17	1.20	[25]
	20	Pet.	48	48, 72	Aluminium patch test chambers mounted on Micropore carried out on the upper back	200	10	5.00	[26]
	20	Pet.	48	48, 72	Finn Chambers on Sanpor applied on the upper back	373	19	5.10	[33]
	20	Pet.	48	48, 96	Finn Chambers on Scanpor tape applied on back	125	8	6.40	[23]
	20	Pet.	48	96	Finn Chambers applied to the back of the patients	618	45	7.30	[44]
	20	Pet.	48	48, 72, 96, 168	Finn Chambers (8 mm), applied on the	3085	318	10.30	[21]

					upper aspect of the back				
**o-Aminophenol**	1	Pet.	48	48, 72, 168	Finn Chambers on Scanpor tape	197	37	18.80	[51]
**p-Aminophenol**	1	Pet.	48	48, 72, 168	Finn Chambers on Scanpor tape	200	21	10.50	[51]
**Paraben Mix**	16	Pet.	48	48, 96	Komory IQ Chambers Chemotechnique Diagnostics	405	1	0.20	[14]
	16	Pet.	48	48, 72, 96, 168	n/a	1993	6	0.30	[24]
	16	Pet.	48	48, 72, 96, 168	n/a	1471	4	0.30	[25]
	16	Pet.	48	48, 72, 96, 168	n/a	1402	4	0.30	[35]
	16	Pet.	48	48, 72, 96, 168	n/a	1399	4	0.30	[35]
	16	Pet.	48	72, 144, 168	IQ Chambers from Chemotechnique Diagnostics AB applied on the back	3112	9	0.30	[32]
	16	Pet.	48	72, 144, 168	IQ Chambers from Chemotechnique Diagnostics AB applied on the back	3825	15	0.40	[32]
	16	Pet.	48	72	Finn Chambers and TRUE Test applied on the back	3662	18	0.50	[32]
	16	Pet.	48	48, 72, 96, 168	Finn Chambers (8mm) on Scanpor tape	18 178	97	0.50	[38]

16	Pet.	48	48, 72	Finn Chambers on Scanpor tape applied to upper back	2758	15	0.50	[27]
16	Pet.	48	48, 96	Finn Chambers applied on Scanpor tape to the upper back of patients	308	2	0.60	[6]
16	Pet.	48	48, 72, 96, 168	n/a	1469	10	0.70	[25]
16	Pet.	48	120, 168	IQ Ultra Chambers applied on the patient's back	1608	11	0.70	[46]
16	Pet.	48	72, 96	n/a	14 728	133	0.90	[10]
16	Pet.	48	48, 96	IQ Chambers	327	3	0.90	[34]
16	Pet.	48	72, 96	n/a	10 124	103	1.00	[10]
16	Pet.	48	48, 72, 96, 168	Finn Chambers on Scanpor tape applied to a patient's upper back	941	9	1.00	[4]
16	Pet.	48	48, 96	Finn Chambers (8 mm) or IQ Chambers	8873	89	1.00	[41]
16	Pet.	48	72, 168	Finn Chambers on Scanpor tape	2598	26	1.00	[47]
16	Pet.	48	72, 96	Curatest Lohman&Rauscher, Rengsdorf Germany	1927	21	1.10	[15]
16	Pet.	48	48, 96	Finn Chambers secured with Scanpor tape and left on the back	3062	34	1.10	[38]
16	Pet.	48	48, 96	Finn Chambers on Scanpor tape to the upper back	6845	75	1.10	[29]

	12	Pet.	48	48, 72, 96, 168	Finn Chambers (8 mm), applied on the upper aspect of the back	4439	53	1.20	[21]
	16	Pet.	48	72, 96	n/a	121 247	1752	1.40	[28]
	16	Pet.	48	48, 72, 96, 168	Finn Chambers (8 mm), applied on the upper back with Scanpor tape	1318	21	1.60	[19]
	16	Pet.	24, 48	72	Finn Chambers (8 mm) on Scan-por (19 departments), Leukotest, Hal, Curatest, Haye, Intradex Service BV, Alphen aan den Rijn,	22 602	364	1.60	[17]
	15	Pet.	48	48, 72	Finn Chambers applied on the upper back	2295	39	1.70	[20]
	16	Pet.	48	48, 72, 96, 168	Finn Chambers (8 mm), applied on the upper back with Scanpor tape	3841	65	1.70	[19]
	16	Pet.	48	48, 72, 96, 168	Finn Chambers (8 mm), applied on the upper aspect of the back	3090	52	1.70	[21]
	16	Pet.	48	48, 72	Aluminium patch test chambers mounted on Micropore carried out on the upper back	200	4	2.00	[26]

	16	Pet.	48	96	Finn Chambers applied to the back of the patients	618	19	3.00	[44]
	16	Pet.	48	48, 96	Finn Chambers (8 mm), applied on the upper back with Scanpor tape	584	18	3.10	[22]
	16	Pet.	48	48, 96	Finn Chambers on Scanpor applied to the upper back	91	3	3.30	[43]
	16	Pet.	48	48, 72	Finn Chambers on Sanpor applied on the upper back	373	19	5.10	[33]
	16	Pet.	48	48, 96	Aluminium chambers carried out on the upper back	33	3	9.10	[45]
**Phenoxyethanol**	1	Pet.	24, 48	72	Finn Chambers (8 mm) on Scan-por (19 departments), Leukotest, Hal, Curatest, Haye, Intradex Service BV, Alphen aan den Rijn,	11 120	14	0.10	[17]
	1	Pet.	48	48, 72, 96, 168	Finn Chambers (8 mm), applied on the upper aspect of the back	2700	3	0.10	[21]
	1	Pet.	48	48, 72, 96, 168	Finn Chambers (8 mm), applied on the upper back with Scanpor tape	4887	10	0.20	[19]

	1	Pet.	48	72, 96	Curatest Lohman&Rauscher, Rengsdorf Germany	1927	6	0.30	[15]
	1	Pet.	48	72, 96	n/a	6932	20	0.30	[28]
	1	Pet.	48	48, 96	Finn Chambers (8 mm), applied on the upper back with Scanpor tape	584	10	1.70	[22]
**p-Phenylenediamine**	1	Pet.	48	48, 72, 96, 168	n/a	1993	24	1.20	[24]
	1	Pet.	48	72	Finn Chambers and TRUE Test applied on the back	3662	51	1.40	[32]
	1	Pet.	48	72, 144, 168	IQ Chambers from Chemotechnique Diagnostics AB applied on the back	3825	77	2.00	[32]
	1	Pet.	48	72, 144, 168	IQ Chambers from Chemotechnique Diagnostics AB applied on the back	3112	65	2.10	[32]
	1	Pet.	48	48, 72, 96, 168	n/a	1396	36	2.60	[35]
	1	Pet.	48	48, 72, 96, 168	n/a	1467	40	2.70	[25]
	1	Pet.	48	48, 96	Finn Chambers secured with Scanpor tape and left on the back	3062	92	3.00	[38]
	1	Pet.	48	48, 72, 96, 168	n/a	1469	44	3.00	[25]
	1	Pet.	48	48, 72, 96, 168	n/a	1397	42	3.00	[35]

	1	Pet.	48	120, 168	IQ Ultra Chambers applied on the patient's back	1608	50	3.10	[46]
	1	Pet.	48	72, 96	n/a	968	37	3.80	[18]
	1	Pet.	48	48, 72	Finn Chambers on Sanpor applied on the upper back	373	16	4.30	[33]
	1	Pet.	48	48, 72, 96, 168	Finn Chambers (8 mm), applied on the upper back with Scanpor tape	3832	172	4.50	[19]
	1	Pet.	48	48, 96	n/a	385	18	4.70	[16]
	1	Pet.	48	48, 72, 96, 168	Finn Chambers (8 mm), applied on the upper back with Scanpor tape	4903	235	4.80	[19]
	1	Pet.	48	48, 72, 96, 168	Finn Chambers (8 mm), applied on the upper back with Scanpor tape	1320	65	4.90	[19]
	1	Pet.	48	48, 72, 96, 168	Finn Chambers (8 mm), applied on the upper aspect of the back	3088	160	5.20	[21]
	1	Pet.	48	48, 72	Finn Chambers on Scanpor tape applied to upper back	2758	144	5.20	[27]
	1	Pet.	48	72, 168	Finn Chambers on Scanpor tape	2598	161	6.20	[47]
	1	Pet.	48	96	Finn Chambers applied to the back of the patients	618	43	7.00	[44]

	1	Pet.	48	48, 72, 96, 168	Finn Chambers (8 mm), applied on the upper aspect of the back	940	69	7.30	[4]
	1	Pet.	48	48, 96	IQ Chambers	327	25	7.60	[34]
	1	Pet.	48	48, 96	n/a	50	5	10.00	[16]
	1	Pet.	48	48, 72	Aluminium patch test chambers mounted on Micropore carried out on the upper back	200	23	11.50	[26]
	1	Pet.	48	72, 96	n/a	457	92	20.10	[30]
	1	Pet.	48	48, 96	Aluminium chambers carried out on the upper back	33	7	21.20	[45]
	1	Pet.	48	72, 96	n/a	1003	244	24.30	[30]
	1	Pet.	48	72, 96	n/a	1003	244	27.00	[30]
	1	Pet.	48	48, 72, 168	Finn Chambers on Scanpor tape	194	68	35.10	[51]
**Potassium dichromate**	0.5	Pet.	48	72, 96	n/a	10 124	367	3.60	[10]
	0.5	Pet.	48	72, 168	Finn Chambers on Scanpor tape	2598	119	4.60	[47]
	0.5	Pet.	48	72, 96	n/a	2044	115	5.60	[37]
	0.5	Pet.	48	48, 72, 96, 168	Finn Chambers (8 mm), applied on the upper back with Scanpor tape	3835	257	6.70	[19]
	0.5	Pet.	48	48, 96	IQ Chambers	327	22	6.70	[34]
	0.5	Pet.	48	48, 96	n/a	385	26	6.80	[16]
	0.5	Pet.	48	48, 72, 96, 168	n/a	1471	43	2.90	[25]

	0.5	Pet.	48	48, 96	n/a	50	1	2.00	[16]
	0.5	Pet.	48	48, 96	Finn Chambers on Scanpor tape applied on back	125	3	2.40	[23]
	0.5	Pet.	48	48, 72, 96, 168	n/a	1398	60	4.30	[35]
	0.5	Pet.	48	72, 144, 168	IQ Chambers from Chemotechnique Diagnostics AB applied on the back	3112	137	4.40	[32]
	0.5	Pet.	48	48, 72, 96, 168	n/a	1468	64	4.40	[25]
	0.5	Pet.	48	72, 96	n/a	14 728	766	5.20	[10]
	0.5	Pet.	48	48, 72	Finn Chambers on Sanpor applied on the upper back	373	27	7.20	[33]
	0.5	Pet.				3092	229	7.40	[21]
	0.5	Pet.	48	96	Finn Chambers applied to the back of the patients	618	69	11.20	[44]
	0.5	Pet.	48	48, 72	Finn Chambers on Scanpor tape applied to upper back	3758	371	13.50	[27]
	0.5	Pet.	48	48, 72	Aluminium patch test chambers mounted on Micropore carried out on the upper back	200	41	20.50	[26]
**Propyl gallate**	1	Pet.	24, 48	72	Finn Chambers (8 mm) on Scan-por (19 departments), Leukotest, Hal,	3133	9	0.30	[17]

					Curatest, Haye, Intradex Service BV, Alphen aan den Rijn,				
	1	Pet.	48	48, 72, 96, 168	Finn Chambers (8 mm), applied on the upper aspect of the back	943	7	0.70	[4]
	1	Pet.	48	48, 96	Finn Chambers (8 mm), applied on the upper back with Scanpor tape	584	4	0.70	[22]
	1	Pet.	48	72, 120	Finn Chambers on Scanpor	41	1	2.40	[21]
	1	Pet.	48	48, 96	Finn Chambers on Scanpor applied to the upper back	91	2	2.20	[43]
**Quaternium 15**	1	Pet.	48	48, 96	Finn Chambers applied on Scanpor tape to the upper back of patients	308	2	0.60	[6]
	1	Pet.	48	72, 96	n/a	6981	43	0.62	[10]
	1	Pet.	24, 48	72	Finn Chambers (8 mm) on Scan-por (19 departments), Leukotest, Hal, Curatest, Haye, Intradex Service BV, Alphen aan den Rijn,	11 017	68	0.62	[17]
	1	Pet.	48	72, 96	n/a	79 745	510	0.64	[28]
	1	Pet.	48	48, 96	Finn Chambers on Scanpor tape	125	1	0.80	[23]
	1	Pet.	48	48, 96	Finn Chambers fixed with Scanpor tape	7838	69	0.88	[35]

	1	Pet.	48	48, 72, 96, 168	n/a	1995	18	0.90	[24]
	1	Pet.	48	48, 72	Finn Chambers applied on the upper back	2295	23	1.00	[20]
	1	Pet.	48	48, 72, 96, 168	Finn Chambers (8mm) on Scanpor tape	18 179	174	1.00	[38]
	1	Pet.	48	72	Finn Chambers and TRUE Test applied on the back	3662	40	1.10	[32]
	1	Pet.	48	48, 96	Komory IQ Chambers Chemotechnique Diagnostics	405	5	1.20	[14]
	1	Pet.	48	72, 144, 168	IQ Chambers from Chemotechnique Diagnostics AB applied on the back	3825	46	1.20	[32]
	1	Pet.	48	48, 72, 96, 168	n/a	1402	17	1.20	[35]
	1	pet	48	48, 96	Finn Chambers secured with Scanpor tape and left on the back	3062	40	1.30	[38]
	1	Pet.	48	72, 144, 168	IQ Chambers from Chemotechnique Diagnostics AB applied on the back	3112	44	1.40	[32]
	1	Pet.	48	48, 72, 96, 168	n/a	1471	20	1.40	[25]

	1	Pet.	48	120, 168	IQ Ultra Chambers applied on the patient's back	1608	26	1.60	[46]
	1	Pet.	48	72, 96	n/a	1735	28	1.60	[37]
	1	Pet.	48	72, 168	Finn Chambers on Scanpor tape	2598	44	1.69	[47]
	1	Pet.	48	48, 96	IQ Chambers	327	6	1.80	[34]
	1	Pet.	48	48, 96	Finn Chambers (8 mm) or IQ Chambers	9361	178	1.90	[41]
	1	Pet.	48	48, 72	Finn Chambers on Sanpor applied on the upper back	373	7	1.90	[33]
	1	Pet.	48	72, 96	Curatest Lohman&Rauscher, Rengsdorf Germany	1927	37	1.90	[15]
	1	Pet.	48	48, 72, 96, 168	n/a	1398	26	1.90	[35]
	1	Pet.	48	48, 72, 96, 168	n/a	1468	29	2.00	[25]
	1	Pet.	48	48, 96	Finn Chambers on Scanpor applied to the upper back	91	2	2.20	[43]
	1	Pet.	48	48, 72	Aluminium patch test chambers mounted on Micropore carried out on the upper back	200	5	2.50	[26]
	1	Pet.	48	96	Finn Chambers applied to the back of the patients	618	16	2.60	[44]
	1	Pet.	48	48, 96	Finn Chambers apllied with Scanpor	2298	59	2.60	[40]

					tape to the upper back				
	1	Pet.	48	48, 96	Finn Chambers on Scanpor tape to the upper back	6845	198	2.90	[29]
	1	Pet.	48	48, 96	Finn Chambers (8 mm), applied on the upper back with Scanpor tape	584	19	3.30	[22]
	2	Pet.	48	48, 72, 96, 168	Finn Chambers (8 mm), applied on the upper back with Scanpor tape	408	21	5.15	[19]
	1	Pet.	48	72, 120	Finn Chambers on Scanpor	34	2	5.90	[21]
	1	Pet.	48	48, 72, 96, 168	Finn Chambers on Scanpor tape applied to a patient's upper back	942	71	7.50	[4]
	1	Pet.	48	48, 72, 96, 168	Finn Chambers (8 mm), applied on the upper back with Scanpor tape	913	71	7.78	[19]
	1	Pet.	48	48, 72, 96, 168	Finn Chambers (8 mm), applied on the upper back with Scanpor tape	3841	311	8.10	[19]
	1	Pet.	48	48, 72, 96, 168	Finn Chambers (8 mm), applied on the upper aspect of the back	3091	275	8.90	[21]
	2	Pet.	48	48, 72, 96, 168	Finn Chambers (8 mm), applied on the	4910	457	9.30	[19]

					upper back with Scanpor tape				
	2	Pet.	48	48, 72, 96, 168	Finn Chambers (8 mm), applied on the upper aspect of the back	4446	458	10.30	[21]
	1	Pet.	48	48, 96	Finn Chambers on Scanpor tape applied to the areas of the back	342	38	11.11	[42]
**Quinoline mix**	6	Pet.	48	72, 144, 168	IQ Chambers from Chemotechnique Diagnostics AB applied on the back	3112	6	0.20	[32]
	6	Pet.	48	72, 168	Finn Chambers on Scanpor tape	2598	10	0.40	[47]
	6	Pet.	48	72, 144, 168	IQ Chambers from Chemotechnique Diagnostics AB applied on the back	3825	19	0.50	[32]
	6	Pet.	48	48, 72, 96, 168	n/a	967	5	0.50	[35]
	6	Pet.	48	48, 72	Finn Chambers on Sanpor applied on the upper back	373	4	1.10	[33]
	6	Pet.	48	48, 96	Finn Chambers on Scanpor tape applied on back	125	2	1.60	[23]
	6	Pet.	48	48, 72, 96, 168	n/a	703	13	1.80	[25]
	6	Pet.	48	48, 72, 96, 168	n/a	789	18	2.30	[25]

	6	Pet.	48	48, 72	Aluminium patch test chambers mounted on Micropore carried out on the upper back	200	5	2.50	[26]
**Sandalwood oil**	2	Pet.	48	48, 96	Finn Chambers applied on Scanpor tape to the upper back of patients	308	2	0.60	[6]
	2	Pet.	48	48, 72, 96, 168	Finn Chambers (8 mm), applied on the upper aspect of the back	870	17	2.00	[4]
	2	Pet.	48	48, 96, 168	Finn Chambers fixed with Scanpor	86	2	2.30	[31]
	2	Pet.	48	n/a	n/a	59	2	3.40	[9]
	10	Pet.	48	48, 120	Finn Chambers (8 mm), applied on the upper back with Scanpor tape	167	11	6.60	[13]
**Sorbic acid**	2	Pet.	48	72, 96	Curatest Lohman&Rauscher, Rengsdorf Germany	1927	4	0.20	[15]
	2	Pet.	48	48, 72, 96, 168	Finn Chambers (8 mm), applied on the upper back with Scanpor tape	610	2	0.30	[19]
	2	Pet.	48	48, 72, 96, 168	Finn Chambers (8 mm), applied on the upper aspect of the back	410	2	0.50	[21]
	2	Pet.	48	72, 96	n/a	77 708	519	0.70	[28]

	2	Pet.	48	72, 96	n/a	6978	47	0.70	[10]
	2	Pet.	24, 48	72	Finn Chambers (8 mm) on Scan-por (19 departments), Leukotest, Hal, Curatest, Haye, Intradex Service BV, Alphen aan den Rijn,	11 437	85	0.70	[17]
	2	Pet.	48	48, 72, 96, 168	Finn Chambers (8 mm), applied on the upper aspect of the back	3084	25	0.80	[21]
	2	Pet.	48	48, 72, 96, 168	Finn Chambers on Scanpor tape applied to a patient's upper back	871	8	0.90	[4]
	2	Pet.	48	48, 96	Finn Chambers (8 mm), applied on the upper back with Scanpor tape	584	12	2.10	[22]
	2	Pet.	48	48, 96	Finn Chambers on Scanpor applied to the upper back	91	4	4.40	[43]
	2	Pet.	48	48, 96	Aluminium chambers carried out on the upper back	33	2	6.10	[45]
**Sorbitan sesquioleate**	20	Pet.	48	48, 72, 96, 168	Finn Chambers (8 mm), applied on the back with Scanpor tape	1502	1	0.10	[2]
	20	Pet.	48	48, 120	Finn Chambers (8 mm), applied on the	167	1	0.60	[13]

					upper back with Scanpor tape				
	20	Pet.	48	48, 96	Finn Chambers applied on Scanpor tape to the upper back of patients	308	2	0.60	[6]
	20	Pet.	48	48, 72, 96, 168	Finn Chambers (8 mm), applied on the upper aspect of the back	870	8	0.90	[4]
	20	Pet.	48	48, 96	Aluminium chambers carried out on the upper back	33	1	3.00	[45]
**Thimerosal**	0.1	Pet	48	48, 96	Finn Chambers applied on Scanpor tape to the upper back of patients	308	5	1.60	[6]
	0.1	Pet	48	48, 72, 96, 168	Finn Chambers (8 mm), applied on the upper back with Scanpor tape	1747	183	10.50	[19]
	0.1	Pet	48	72, 96	Curatest Lohman&Rauscher, Rengsdorf Germany	1927	218	11.30	[15]
	0.1	Pet	48	48, 72	Finn Chambers on Scanpor tape applied to upper back	2758	320	11.60	[27]
	0.1	Pet	48	48, 72, 96, 168	Finn Chambers (8 mm), applied on the upper aspect of the back	942	128	13.60	[4]

	0.1	Pet	48	96	Finn Chambers applied to the back of the patients	618	99	16.00	[44]
	0.1	Pet	48	48, 96	Finn Chambers on Scanpor tape applied on back	125	23	18.40	[23]
	0.1	Pet	48	72, 96	n/a	235	48	20.40	[37]
	0.1	Pet	48	48, 96	Aluminium chambers carried out on the upper back	33	1	3.00	[45]
	0.1	Pet	48	48, 96	n/a	50	2	4.00	[16]
	0.1	Pet	48	48, 72	Finn Chambers applied on the upper back	2295	96	4.20	[20]
	0.1	Pet	48	48, 96	n/a	385	21	5.50	[16]
	0.1	Pet	48	48, 72	Finn Chambers on Sanpor applied on the upper back	373	28	7.50	[33]
	0.1	Pet	48	48, 96	Finn Chambers (8 mm), applied on the upper back with Scanpor tape	584	58	9.90	[22]
**Thiuram mix**	1	Pet.	48	72, 144, 168	IQ Chambers from Chemotechnique Diagnostics AB applied on the back	3112	50	1.60	[32]
	1	Pet.	48	48, 72	Finn Chambers on Sanpor applied on the upper back	373	10	2.70	[33]
	1	Pet.	48	72, 96	n/a	2026	157	7.70	[37]
	1	Pet.	48	72, 96	n/a	1905	37	1.90	[30]
	1	Pet.	48	72, 96	n/a	14 728	280	1.90	[10]

	1	Pet.	48	72, 120	Finn Chambers on Scanpor	34	1	2.90	[21]
	1	Pet.	48	72, 96	n/a	698	20	2.90	[30]
	1	Pet.	48	48, 96	Finn Chambers on Scanpor tape applied on back	125	0	0.00	[23]
	1	Pet.	48	72, 168	Finn Chambers on Scanpor tape	2598	5	0.20	[47]
	1	Pet.	48	48, 96	IQ Chambers	327	1	0.30	[34]
	1	Pet.	48	72, 96	n/a	10 124	127	1.20	[10]
	1	Pet.	48	72	Finn Chambers and TRUE Test applied on the back	3662	73	2.00	[32]
	1	Pet.	48	48, 72, 96, 168	n/a	1468	36	2.50	[25]
	1	Pet.	48	48, 72, 96, 168	n/a	1471	37	2.50	[25]
	1	Pet.	48	48, 72, 96, 168	Finn Chambers (8 mm), applied on the upper aspect of the back	3094	96	3.10	[21]
	1	Pet.	48	48, 72, 96, 168	Finn Chambers (8 mm), applied on the upper back with Scanpor tape	3838	127	3.30	[19]
	1	Pet.	48	48, 72, 96, 168	n/a	1398	48	3.40	[35]
	1	Pet.	48	48, 96	n/a	385	13	3.40	[16]
	1	Pet.	48	48, 72	Aluminium patch test chambers mounted on Micropore carried	200	9	4.50	[26]

					out on the upper back				
	1	Pet.	48	96	Finn Chambers applied to the back of the patients	618	38	6.20	[44]
	1	Pet.	48	48, 96	Finn Chambers on Scanpor applied to the upper back	91	10	11.00	[43]
**Titanium Dioxide**	1	Pet.	48	48, 72, 96, 168	Finn Chambers (8 mm), applied on the upper aspect of the back	921	8	0.90	[4]
**Toluene-2,5-diamine sulfate**	1	Pet.	48	48, 72, 168	Finn Chambers on Scanpor tape	198	23	11.60	[51]
**Triethanolamine**	2	Pet.	48	48, 72, 96, 168	Finn Chambers (8 mm), applied on the upper aspect of the back	944	6	0.60	[4]
	2	Pet.	48	48, 72, 96, 168	Finn Chambers (8 mm), applied on the upper back with Scanpor tape	3845	31	0.80	[19]
	2	Pet.	48	48, 96	Finn Chambers (8 mm), applied on the upper back with Scanpor tape	584	21	3.60	[22]
**Wool alcohols**	30	Pet.	48	48, 72, 96, 168	Finn Chambers (8 mm), applied on the upper aspect of the back	942	17	1.80	[4]
	30	Pet.	48	48, 72, 96, 168	Finn Chambers (8 mm), applied on the	3842	73	1.90	[19]

					upper back with Scanpor tape				
	30	Pet.	48	48, 72	Finn Chambers on Sanpor applied on the upper back	373	9	2.40	[33]
	30	Pet.	48	48, 72, 96, 168	Finn Chambers (8 mm), applied on the upper aspect of the back	3032	73	2.40	[21]
	30	Pet.	48	48, 96	Finn Chambers on Scanpor applied to the upper back	91	7	7.70	[43]
**Ylang Ylang oil**	2	Pet.	48	48, 72, 96, 168	Finn Chambers (8 mm), applied on the upper aspect of the back	870	8	0.90	[4]
	2	Pet.	48	48, 72, 96, 168	Finn Chambers (8 mm), applied on the upper back with Scanpor tape	4893	54	1.10	[19]
	2	Pet.	48	120, 168	IQ Ultra Chambers applied on the patient's back	1608	17	1.10	[46]
	2	Pet.	48	72, 96	n/a	917	23	2.50	[37]
	2	Pet.	48	48, 96, 168	Finn Chambers fixed with Scanpor	86	12	13.90	[31]
	10	Pet.	48	48, 120	Finn Chambers (8 mm), applied on the upper back with Scanpor tape	167	29	17.40	[13]
	2	Pet.	48	n/a	n/a	59	16	27.10	[9]

Table S2. Summary of patch test studies in general populations.

Name of ingredient	Concentration (%)	Base	Occlusion (h)	Time to evaluate reactions	Application system	N	n	n (%)	Source
**Ammylcinnamyl alcohol**	2	Pet.	n/a	n/a	TRUE test and Finn Chambers	3117	3	0.10	[5]
**Balsam of Peru**	25	Pet.	48	48	TRUE test	3460	3	0.10	[52]
	25	Pet.	48	72	TRUE test applied to the upper back	1236	5	0.40	[53]
	25	Pet.	n/a	n/a	TRUE test and Finn Chambers	3117	23	0.70	[5]
	25	Pet.	48	48, 72, 96, 168	n/a	399	3	0.80	[24]
	25	Pet.	48	72	TRUE test applied to the upper back	531	6	1.10	[53]
	25	Pet.	48	48	TRUE test applied to the upper back	567	6	1.10	[54]

	25	Pet.	48	48	TRUE test	543	6	1.10	[52]
	25	Pet.	48	48	TRUE test applied to the upper back	469	6	1.30	[55]
	25	Pet.	48	48, 72, 96, 168	n/a	1471	35	2.40	[25]
	25	Pet.	48	48, 72, 96, 168	n/a	1400	43	3.10	[35]
	25	Pet.	48	72	Finn Chambers applied on the upper back	1141	43	3.80	[56]
**Benzocaine**	5	Pet.	48	48, 72, 96, 168	n/a	698	1	0.10	[35]
	5	Pet.	48	72	Finn Chambers applied on the upper back	1141	11	1.00	[56]
	5	Pet.	48	48, 72, 96, 168	n/a	318	4	1.30	[24]
**Benzyl alcohol**	1	Pet.	48	48, 96	TRUE test applied to the upper back	201	0	0.00	[57]
**Benzyl salicylate**	1	Pet.	48	48, 96	TRUE test applied to the upper back	201	1	0.50	[57]
**Black rubber mix**	0.6	Pet.	48	48	TRUE test	3460	4	0.10	[52]
	0.6	Pet.	48	72	TRUE test applied to the upper back	1236	1	0.10	[53]
	0.6	Pet.	48	48	TRUE test applied to the upper back	567	1	0.20	[54]
	0.6	Pet.	48	48	TRUE test	567	1	0.20	[52]
	0.6	Pet.	n/a	n/a	TRUE test and Finn Chambers	3117	11	0.40	[5]
	0.6	Pet.	48	48, 96	IQ Chambers	205	1	0.50	[34]
**Bronopol**	0.5	Pet.	48	48, 96	IQ Chambers	205	0	0.00	[34]
	0.5	Pet.	48	48, 96	TRUE test applied to the upper back	201	0	0.00	[57]
	0.5	Pet.	48	48, 72, 96, 168	n/a	1389	4	0.30	[35]

**Butylhydroxyanisole (BHA)**	2	Pet.	48	48, 96	TRUE test applied to the upper back	201	2	2.20	[57]
**Caine mix**	7	Pet.	48	48	TRUE test applied to the upper back	567	0	0.00	[54]
	7	Pet.	48	48	TRUE test applied to the upper back	290	0	0.00	[55]
	7	Pet.	48	48	TRUE test	567	0	0.00	[52]
	7	Pet.	48	48	TRUE test	3460	2	0.10	[52]
	7	Pet.	48	72	TRUE test applied to the upper back	1236	1	0.10	[53]
	7	Pet.	48	48	TRUE test applied to the upper back	469	1	0.20	[55]
	7	Pet.	n/a	n/a	TRUE test and Finn Chambers	3119	13	0.40	[5]
	7	Pet.	48	72	TRUE test applied to the upper back	531	2	0.40	[53]
**Carba mix**	3	Pet.	48	48	TRUE test	567	4	0.20	[52]
	3	Pet.	48	48	TRUE test applied to the upper back	567	2	0.40	[54]
	3	Pet.	n/a	n/a	TRUE test and Finn Chambers	3117	17	0.50	[5]
	3	Pet.	48	72	TRUE test applied to the upper back	1236	8	0.60	[53]
	3	Pet.	48	48, 96	IQ Chambers	205	5	2.40	[34]
**Cinnamal**	1	Pet.	n/a	n/a	TRUE test and Finn Chambers	3117	26	0.80	[5]
**Cinnamic alcohol**	1	Pet.	n/a	n/a	TRUE test and Finn Chambers	3117	10	0.30	[5]
**Citral**	2	Pet.	n/a	n/a	TRUE test and Finn Chambers	3117	6	0.20	[5]
**Cobalt (di)chloride**	1	Pet.	48	48	TRUE test applied to the upper back	567	6	1.10	[54]
	1	Pet.	48	72	Finn Chambers applied on the upper back	1141	27	2.40	[56]

	1	Pet.	48	48, 72, 96, 168	n/a	399	18	4.50	[24]
	1	Pet.	48	48, 96	IQ Chambers	205	10	4.90	[34]
	1	Pet.	48	48, 72, 96, 168	n/a	1400	74	5.30	[35]
	1	Pet.	48	48	TRUE test applied to the upper back	469	4	0.80	[55]
	1	Pet.	48	48	TRUE test applied to the upper back	290	3	1.00	[55]
	1	Pet.	48	48	TRUE test	567	6	1.10	[52]
	1	Pet.	48	72	TRUE test applied to the upper back	531	9	1.70	[53]
	1	Pet.	n/a	n/a	TRUE test and Finn Chambers	3117	68	2.20	[5]
	1	Pet.	48	72	TRUE test applied to the upper back	1236	35	2.80	[53]
**Cocamidopropyl betaine**	1	Aq.	48	48, 96	TRUE test applied to the upper back	201	4	2.00	[57]
**Colophonium**	20	Pet.	48	48	TRUE test applied to the upper back	290	0	0.00	[55]
	20	Pet.	48	48	TRUE test	3460	21	0.60	[52]
	20	Pet.	48	48	TRUE test applied to the upper back	567	4	0.70	[54]
	20	Pet.	48	48	TRUE test	543	4	0.70	[52]
**Colophonium – continued**	20	Pet.	48	48	TRUE test applied to the upper back	469	4	0.80	[55]
	20	Pet.	n/a	n/a	TRUE test and Finn Chambers	3117	30	1.00	[5]
	20	Pet.	48	72	TRUE test applied to the upper back	531	6	1.10	[53]
	20	Pet.	48	72	TRUE test applied to the upper back	1236	15	1.20	[53]
	20	Pet.	48	48, 96	IQ Chambers	205	3	1.50	[34]

	20	Pet.	48	48, 72, 96, 168	n/a			1.50	[24]
	20	Pet.	48	72	Finn Chambers applied on the upper back	1141	18	1.60	[56]
	20	Pet.	48	48	Finn Chambers using Scanpor adhesive tape on the upper back	1178	24	2.00	[58]
	20	Pet.	48	72, 96	n/a	14 728	574	3.90	[10]
	20	Pet.	48	48, 72, 96, 168	n/a	1470	64	4.40	[25]
	20	Pet.	48	48, 72, 96, 168	n/a	1400	64	4.60	[35]
**Diazolidynyl Urea**	2	Pet.	n/a	n/a	TRUE test and Finn Chambers	3117	6	0.20	[5]
	2	Pet.	48	48, 96	TRUE test applied to the upper back	201	1	0.5	[57]
	2	Pet.	48	48, 72, 96, 168	n/a	1398	15	1.10	[35]
**DMDM Hydantoin**	2	Aq.	48	48, 96	TRUE test applied to the upper back	201	0	0.00	[57]
**Epoxy resin**	1	Pet.	48	48	TRUE test applied to the upper back	290	0	0.00	[55]
	1	Pet.	48	48	TRUE test applied to the upper back	469	1	0.20	[55]
	1	Pet.	48	48	TRUE test	3460	18	0.50	[52]
	1	Pet.	48	48	TRUE test applied to the upper back	567	3	0.50	[54]
	1	Pet.	48	48, 96	IQ Chambers	205	1	0.50	[34]
	1	Pet.	48	72	Finn Chambers	1141	7	0.60	[56]

	1	Pet.	48	72	TRUE test applied to the upper back	1236	7	0.60	[53]
	1	Pet.	48	48	TRUE test	567	3	0.60	[52]
	1	Pet.	48	72	TRUE test applied to the upper back	531	4	0.80	[53]
	1	Pet.	n/a	n/a	TRUE Test and Finn Chambers	3117	28	0.90	[5]
	1	Pet.	48	48, 72, 96, 168	n/a	1401	19	1.40	[35]
**Ethylenediamine (dichloride)**	1	Pet.	48	48	TRUE test applied to the upper back	567	1	0.20	[54]
	1	Pet.	48	72	TRUE test applied to the upper back	1236	1	0.10	[53]
	1	Pet.	48	48	TRUE test	3460	8	0.20	[52]
	1	Pet.	48	48	TRUE test	567	1	0.20	[52]
	1	Pet.	48	48	TRUE test applied to the upper back	290	1	0.30	[55]
	1	Pet.	n/a	n/a	TRUE Test and Finn Chambers	3117	11	0.40	[5]
	1	Pet.	48	48	TRUE test applied to the upper back	469	5	1.10	[55]
	1	Pet.	48	48, 96	IQ Chambers	205	3	1.50	[34]
**Evernia Prunastri Extract**	2	Pet.	n/a	n/a	TRUE Test and Finn Chambers	3117	16	0.50	[5]
	2	Pet.	n/a	n/a	TRUE Test and Finn Chambers	3117	31	1.00	[5]
**Farnesol**	5	Pet.	n/a	n/a	TRUE test and Finn Chambers	3117	14	0.40	[5]
**Formaldehyde**	1	Pet.	48	48	TRUE test	3460	7	0.20	[52]
	1	Pet.	n/a	n/a	TRUE test and Finn Chambers	3117	11	0.35	[5]
	1	Pet.	48	72	Finn Chambers	1141	7	0.61	[56]

	1	Pet.	48	48	TRUE test applied to the upper back	469	3	0.64	[55]
	1	Pet.	48	48	Finn Chambers using Scanpor adhesive tape on the upper back	2545	19	0.75	[58]
	1	Pet.	48	72	TRUE test applied to the upper back	1236	11	0.90	[53]
	1	Aq.	48	48, 72, 96, 168	n/a	399	6	1.50	[24]
	1	Aq.	48	48, 72, 96, 168	n/a	1397	27	1.90	[35]
	1	Aq.	48	48, 72, 96, 168	n/a	1395	35	2.50	[35]
	1	Aq.	48	48, 72, 96, 168	n/a	1471	43	3.00	[25]
**Fragrance Mix I**	8	Pet.	48	48	TRUE test applied to the upper back	290	1	0.30	[55]
	8	Pet.	48	48	TRUE test applied to the upper back	567	6	1.10	[54]
	8	Pet.	48	48	TRUE test	543	6	1.10	[52]
	8	Pet.	n/a	n/a	TRUE test and Finn Chambers	3117	50	1.60	[5]
	8	Pet.	48	48	TRUE test	3460	55	1.60	[52]
	8	Pet.	48	72	TRUE test applied to the upper back	1236	22	1.80	[53]
	8	Pet.	48	48	TRUE test applied to the upper back	469	11	2.30	[55]
	8	Pet.	48	48, 96	IQ Chambers	205	5	2.40	[34]
	8	Pet.	48	48	Finn Chambers using Scanpor adhesive tape on the upper back	2545	63	2.50	[58]
	8	Pet.	n/a	n/a	TRUE test and Finn Chambers	3117	82	2.60	[5]

	8	Pet.	48	72	TRUE test applied to the upper back	531	18	3.40	[53]
	8	Pet.	48	48, 72, 96, 168	n/a	399	18	4.50	[24]
	8	Pet.	48	72, 96	n/a	14 728	869	5.90	[10]
	8	Pet.	48	48, 72, 96, 168	n/a	1396	107	7.70	[35]
	8	Pet.	48	48, 72, 96, 168	n/a	1470	115	7.80	[25]
	8	Pet.	48	72	Finn Chambers applied on the upper back	1141	181	15.90	[56]
**Fragrance Mix II**	14	Pet.	n/a	n/a	TRUE test and Finn Chambers	3117	60	0.90	[5]
**Geraniol**	2	Pet.	48	n/a	TRUE Test and Finn Chambers	3117	13	0.40	[5]
**Hydroxycitronellal**	2	Pet.	48	n/a	TRUE Test and Finn Chambers	3117	15	0.50	[5]
**Imidiazolidynyl urea**	2	Pet.	n/a	n/a	TRUE test and Finn Chambers	3117	6	0.20	[5]
	2	Pet.	48	48, 96	IQ Chambers	205	1	0.50	[34]
	2	Pet.	48	48, 96	TRUE test applied to the upper back	201	1	0.50	[57]
	2	Pet.	48	48, 72, 96, 168	n/a	1398	8	0.60	[35]
**Iodopropynyl butylcarbamate**	0.2	Pet.	48	48, 96	TRUE test applied to the upper back	201	1	0.50	[57]
	0.2	Pet.	48	48, 72, 96, 168	n/a	1451	8	0.60	[25]
	0.2	Pet.	48	48, 72, 96, 168	n/a	1395	8	0.60	[35]
	0.2	Pet.	48	48, 72, 96, 168	n/a	1390	10	0.70	[35]
**Isopropyl myristate**	20	Pet.	48	48, 96	TRUE test applied to the upper back	201	0	0.00	[57]

**Lanolin**	30	Pet.	48	48, 72, 96, 168	n/a	1401	8	0.60	[35]
	30	Pet.	48	48, 72, 96, 168	n/a	399	5	1.30	[24]
**Hydroxyisohexyl 3-cyclohexene carboxaldehyde (HICC) - Lyral**	5	Pet.	n/a	n/a	TRUE test and Finn Chambers	3117	45	1.40	[5]
	5	Pet.	48	48, 72, 96, 168	n/a	398	8	2.00	[24]
	5	Pet.	48	48, 72, 96, 168	n/a	1398	55	4.00	[35]
**Methylchloroisothiazolinone**	0.01	Aq.	48	48, 96	IQ Chambers	205	0	0.00	[34]
	0.01	Aq.	48	48	TRUE test	3460	6	0.20	[52]
	0.01	Aq.	48	72	TRUE test applied to the upper back	1236	8	0.60	[53]
**Methylchloroisothiazolinone/ Methylisothiazolinone**	0.01	Aq.	48	48	TRUE test applied to the upper back	290	0	0.00	[55]
	0.01	Aq.	48	48	TRUE test applied to the upper back	469	1	0.20	[55]
	0.01	Aq.	n/a	n/a	Finn Chambers on Scanpor	593	1	0.20	[58]
	0.01	Aq.	n/a	n/a	TRUE test and Finn Chambers	3117	15	0.50	[5]
	0.02	Aq.	48	48, 96	TRUE test applied to the upper back	201	1	0.50	[57]
	0.01	Aq.	48	48	Finn Chambers using Scanpor adhesive tape on the upper back	1178	14	1.20	[58]
**Methylchloroisothiazolinone/ Methylisothiazolinone – continued**	0.01	Aq.	48	48, 72, 96, 168	n/a	399	7	1.80	[24]
	0.01	Aq.	48	48, 72, 96, 168	n/a	1395	58	4.20	[35]
**Methylisothiazolinone**	0.20	Aq.	48	48, 96	TRUE test applied to the upper back	201	1	0.50	[57]
	0.05	Aq.	48	48, 72, 96, 168	n/a	1060	25	2.40	[35]

**Methyldibromo Glutaronitrile (MDBGN)**	0.30	Pet.	48	48, 96	TRUE test applied to the upper back	201	1	0.50	[57]
	0.30	Pet.	48	48, 72, 96, 168	n/a	390	6	1.50	[24]
	0.30	Pet.	48	72	Finn Chambers	1141	19	1.70	[56]
	0.30	Pet.	48	48, 72, 96, 168	n/a	1392	46	3.30	[35]
**Mercaptobenzothiazole**	2.0	Pet.	48	48	TRUE test	3460	0	0.00	[52]
	2.0	Pet.	48	72	TRUE test applied to the upper back	1236	1	0.10	[53]
	2.0	Pet.	48	48	TRUE test applied to the upper back	567	1	0.20	[54]
	2.0	Pet.	48	72	TRUE test applied to the upper back	567	1	0.20	[52]
	2.0	Pet.	48	48, 72, 96, 168	n/a	399	1	0.30	[24]
	2.0	Pet.	48	72	Finn Chambers	1141	3	0.30	[56]
	2.0	Pet.	48	48, 72, 96, 168	n/a	1402	9	0.60	[35]
**Mercapto mix**	2.0	Pet.	48	48	TRUE test	3460	0	0.00	[52]
	2.0	Pet.	n/a	n/a	TRUE test and Finn Chambers	3117	7	0.20	[5]
	2.0	Pet.	48	72	TRUE test applied to the upper back	1236	3	0.20	[53]
	2.0	Pet.	48	48	TRUE test applied to the upper back	469	1	0.20	[55]
	2.0	Pet.	48	72	Finn Chambers	1141	3	0.3	[56]
	2.0	Pet.	48	48, 72, 96, 168	n/a	399	1	0.30	[24]
	2.0	Pet.	48	48	TRUE test applied to the upper back	567	2	0.40	[54]
	2.0	Pet.	48	48	TRUE test	567	2	0.40	[52]
	2.0	Pet.	48	48	TRUE test applied to the upper back	290	2	0.70	[55]

	2.0	Pet.	48	48, 72, 96, 168	n/a	1402	12	0.90	[35]
	2.0	Pet.	48	48, 96	IQ Chambers	205	3	1.50	[34]
**Neomycin sulfate**	20	Pet.	48	48	TRUE test applied to the upper back	567	0	0.00	[54]
	20	Pet.	48	48	TRUE test	567	0	0.00	[52]
	20	Pet.	48	48	TRUE test	3460	2	0.10	[52]
	20	Pet.	n/a	n/a	TRUE test and Finn Chambers	3117	11	0.40	[5]
	20	Pet.	48	72	TRUE test applied to the upper back	1236	6	0.50	[53]
	20	Pet.	48	48, 72, 96, 168	n/a	1400	17	1.20	[35]
	20	Pet.	48	72	Finn Chambers	1141	16	1.40	[56]
**Paraben Mix**	16	Pet.	48	48, 96	IQ Chambers	205	0	0.00	[34]
	16	Pet.	48	48, 72, 96, 168	n/a	399	0	0.00	[24]
	16	Pet.	48	48	TRUE test	3460	0	0.00	[52]
	16	Pet.	n/a	n/a	TRUE test and Finn Chambers	3117	3	0.10	[5]
	16	Pet.	48	72	TRUE test applied to the upper back	1236	1	0.10	[53]
	16	Pet.	48	48, 72, 96, 168	n/a	1402	4	0.30	[35]
	16	Pet.	48	48	TRUE test applied to the upper back	567	2	0.40	[54]
	16	Pet.	48	48	TRUE test	567	2	0.40	[52]
	16	Pet.	48	48, 96	TRUE test applied to the upper back	201	1	0.50	[57]
	16	Pet.	48	72	Finn Chambers	1141	7	0.60	[56]
	16	Pet.	n/a	n/a	Finn Chambers on Scanpor	593	4	0.70	[58]
**p-Phenylenediamine**	1	Pet.	48	48, 96	IQ Chambers	205	0	0.00	[34]
	1	Pet.	48	48	TRUE test applied to the upper back	567	0	0.00	[54]

	1	Pet.	48	48	TRUE test applied to the upper back	290	0	0.00	[55]
	1	Pet.	48	48	TRUE test	567	0	0.00	[52]
	1	Pet.	48	48	TRUE test	3460	4	0.10	[52]
	1	Pet.	48	48	TRUE test applied to the upper back	469	1	0.20	[55]
	1	Pet.	48	72	TRUE test applied to the upper back	1236	4	0.30	[53]
	1	Pet.	n/a	n/a	Finn Chambers on Scanpor	593	3	0.50	[58]
	1	Pet.	n/a	n/a	TRUE test and Finn Chambers	3117	30	1.00	[5]
	1	Pet.	48	72	Finn Chambers and TRUE Test applied on the back	3662	51	1.40	[32]
	1	Pet.	48	72	Finn Chambers applied on the upper back	1141	17	1.50	[56]
	1	Pet.	48	72, 144, 168	IQ Chambers from Chemotechnique Diagnostics AB applied on the back	3825	77	2.00	[32]
	1	Pet.	48	72, 144, 168	IQ Chambers from Chemotechnique Diagnostics AB applied on the back	3112	65	2.10	[32]
	1	Pet.	48	48, 72, 96, 168	n/a	1396	36	2.60	[35]
	1	Pet.	48	48	Finn Chambers using Scanpor adhesive tape on the upper back	2545	69	2.70	[58]
	1	Pet.	48	48, 96	Finn Chambers secured with Scanpor tape and left on the back	3062	92	3.00	[39]
	1	Pet.	48	48, 72, 96, 168	n/a	399	36	9.00	[24]
**Potassium dichromate**	0.50	Pet.	48	48	TRUE test	3460	5	0.10	[52]

	0.50	Pet.	48	48	TRUE test applied to the upper back	290	1	0.30	[55]
	0.50	Pet.	48	48	TRUE test applied to the upper back	567	3	0.50	[54]
	0.50	Pet.	48	48	TRUE test	567	3	0.60	[52]
	0.50	Pet.	n/a	n/a	TRUE test and Finn Chambers	3117	25	0.80	[5]
	0.50	Pet.	48	72	TRUE test applied to the upper back	1236	10	0.80	[53]
	0.50	Pet.	48	72	TRUE test applied to the upper back	531	4	0.80	[53]
	0.50	Pet.	48	72	Finn Chambers applied on the upper back	1141	12	1.10	[56]
	0.50	Pet.	48	48	TRUE test applied to the upper back	469	5	1.10	[55]
	0.50	Pet.	48	48, 72, 96, 168	n/a	1401	39	2.80	[35]
	0.50	Pet.	48	48, 96	IQ Chambers	205	5	2.40	[34]
**Propylene glycol**	5	Pet.	48	48, 96	TRUE test applied to the upper back	201	0	0.00	[57]
**Propyl gallate**	1	Pet.	48	48, 96	TRUE test applied to the upper back	201	0	0.00	[57]
**Sorbitan sesquioleate**	20	Pet.	48	48, 96	TRUE test applied to the upper back	201	1	0.50	[57]
**Thimerosal**	0.10	Pet	48	48	TRUE test	3460	18	0.50	[52]
	0.10	Pet	48	72	TRUE test applied to the upper back	1236	23	1.90	[53]
	0.10	Pet	48	48, 96	TRUE test applied to the upper back	201	39	19.40	[57]
	0.10	Pet	48	48	TRUE test applied to the upper back	469	10	2.10	[55]
	0.10	Pet	48	48	TRUE test applied to the upper back	290	8	2.80	[55]
	0.10	Pet	48	48	TRUE test applied to the upper back	567	19	3.40	[54]
	0.10	Pet	48	48	TRUE test	567	19	3.50	[52]

	0.10	Pet	48	72	Finn Chambers applied on the upper back	1141	54	4.70	[56]
	0.10	Pet	n/a	n/a	TRUE test and Finn Chambers	3117	155	5.00	[5]
**Thiuram mix**	1	Pet.	48	48, 96	IQ Chambers	205	0.00	0.00	[34]
	1	Pet.	48	48	TRUE test applied to the upper back	290	0.00	0.00	[55]
	1	Pet.	48	48	TRUE test	3460	3	0.10	[52]
	1	Pet.	48	48	TRUE test applied to the upper back	469	2	0.40	[55]
	1	Pet.	n/a	n/a	TRUE test and Finn Chambers	3117	15	0.50	[5]
	1	Pet.	48	48	TRUE test applied to the upper back	567	3	0.50	[54]
	1	Pet.	48	72	TRUE test applied to the upper back	1236	7	0.60	[53]
	1	Pet.	48	48	TRUE test	567	3	0.60	[52]
	1	Pet.	48	72	Finn Chambers applied on the upper back	1141	8	0.70	[56]
	1	Pet.	48	48, 72, 96, 168	n/a	399	10	2.50	[24]
	1	Pet.	48	48, 72, 96, 168	n/a	1401	75	5.40	[35]
**Triethanolamine**	2	Pet.	48	48, 96	TRUE test applied to the upper back	201	0	0.00	[57]
**Sorbitan sesquioleate**	20	Pet.	48	48, 96	TRUE test applied to the upper back	201	1	0.50	[57]
**Quaternium 15**	1	Pet.	48	48	TRUE test	543	1	0.20	[52]
	1	Pet.	48	48	TRUE test applied to the upper back	567	1	0.20	[54]
	1	Pet.	48	48	TRUE test applied to the upper back	469	1	0.20	[55]
	1	Pet.	48	48	TRUE test	3460	6	0.20	[52]
	1	Pet.	n/a	n/a	TRUE test and Finn Chambers	3117	12	0.40	[5]

	1	Pet.	48	72	TRUE test applied to the upper back	1236	5	0.40	[53]
	1	Pet.	48	48, 96	TRUE test applied to the upper back	201	1	0.50	[57]
	1	Pet.	48	48, 72, 96, 168	n/a	399	4	1.00	[24]
	1	Pet.	48	48, 72, 96, 168	n/a	1402	17	1.20	[35]
	1	Pet.	48	48, 72, 96, 168	n/a	1471	20	1.40	[25]
	1	Pet.	48	48, 72, 96, 168	n/a	1398	26	1.90	[35]
**Quinoline mix**	6	Pet.	48	48	TRUE test	3460	2	0.10	[52]
	6	Pet.	48	72	TRUE test applied to the upper back	1236	1	0.10	[53]
	6	Pet.	n/a	n/a	TRUE test and Finn Chambers	3117	5	0.20	[5]
	6	Pet.	48	48	TRUE test applied to the upper back	567	2	0.40	[54]
	6	Pet.	48	48	TRUE test	567	2	0.40	[52]
	6	Pet.	48	72	TRUE test applied to the upper back	531	3	0.60	[53]
	6	Pet.	48	48, 72, 96, 168	n/a	702	5	0.70	[35]
**Wool Acohols**	30	Pet.	48	72	TRUE test applied to the upper back	1236	1	0.10	[53]
	30	Pet.	48	48	TRUE test applied to the upper back	567	1	0.20	[54]
	30	Pet.	48	48	TRUE test	567	1	0.20	[52]
	30	Pet.	48	48	TRUE test applied to the upper back	469	1	0.20	[55]
	30	Pet.	48	48	TRUE test applied to the upper back	290	1	0.30	[55]
	30	Pet.	n/a	n/a	TRUE test and Finn Chambers	3119	14	0.40	[5]

30	Pet.	48	72	Finn Chambers applied on the upper back	1141	16	1.40	[56]

Table S3. Selected descriptors which are a key to discrimination of molecules according to their sensitizing effects.

eAR		eOR	
**Descriptor**	**Explanation**	**Descriptor**	**Explanation**
**AATS6p**	Average Broto-Moreau autocorrelation - lag 6 / weighted by polarizabilities	AATS6m	Average Broto-Moreau autocorrelation - lag 6 / weighted by mass
**ATSC5v**	Centered Broto-Moreau autocorrelation - lag 5 / weighted by van der Waals volumes	AATS8m	Average Broto-Moreau autocorrelation - lag 8 / weighted by mass
**ATSC3p**	Centered Broto-Moreau autocorrelation - lag 3 / weighted by polarizabilities	ATSC7m	Centered Broto-Moreau autocorrelation - lag 7 / weighted by mass
**AATSC3v**	Average centered Broto-Moreau autocorrelation - lag 3 / weighted by van der Waals volumes	AATSC7m	Average centered Broto-Moreau autocorrelation - lag 7 / weighted by mass
**AATSC3p**	Average centered Broto-Moreau autocorrelation - lag 3 / weighted by polarizabilities	GATS3s	Geary autocorrelation - lag 3 / weighted by I-state
**MATS3v**	Moran autocorrelation - lag 3 / weighted by van der Waals volumes	nHAvin	Count of atom-type H E-State: H on C vinyl bonded to C aromatic

**MATS2p**	Moran autocorrelation - lag 2 / weighted by polarizabilities	naaaC	Count of atom-type E-State: ::C:
**MATS3p**	Moran autocorrelation - lag 3 / weighted by polarizabilities	naaN	Count of atom-type E-State: :NH:
**GATS3p**	Geary autocorrelation - lag 3 / weighted by polarizabilities	SaaaC	Sum of atom-type E-State: ::C:
**GATS4p**	Geary autocorrelation - lag 4 / weighted by polarizabilities	SaaN	Sum of atom-type E-State: :NH:
**GATS2i**	Geary autocorrelation - lag 2 / weighted by first ionization potential	minaaaC	Minimum atom-type E-State: ::C:
**VC-3**	Valence cluster, order 3	minaaN	Minimum atom-type E-State: :NH:
**VC-4**	Valence cluster, order 4	maxaaaC	Maximum atom-type E-State: ::C:
**naaaC**	Count of atom-type E-State: ::C:	maxaaN	Maximum atom-type E-State: :NH:
**naaN**	Count of atom-type E-State: :NH:	piPC6	Conventional bond order ID number of order 6 (ln(1+x)
**SHAvin**	Sum of atom-type H E-State: H on C vinyl bonded to C aromatic	piPC7	Conventional bond order ID number of order 7 (ln(1+x)
**minHdsCH**	Minimum atom-type H E-State: =CH-	SRW5	Self-returning walk count of order 5 (ln(1+x)
**minHAvin**	Minimum atom-type H E-State: H on C vinyl bonded to C aromatic		
**maxHaaCH**	Maximum atom-type H E-State: :CH:		
**maxHAvin**	Maximum atom-type H E-State: H on C vinyl bonded to C aromatic		
**ETA_Shape_X**	Shape index X		
**ETA_Beta_ns_d**	A measure of lone electrons entering into resonance		
**JGI1**	Mean topological charge index of order 1		
**JGI9**	Mean topological charge index of order 9		

Table S4. Comparison of LLNA and in vitro test results of cosmetic ingredients and topical drugs with predicted results by the in silico model.

		Present study				Published data	
**Substance**	**Hapten**	**eAR**	**In silico**	**eOR**	**In silico**	**LLNA**	**In vitro**
**Ammylcinnamyl alcohol**	Ammylcinnamyl alcohol	0	1	1	1	n/a	n/a
**Myroxylon pereirae resin**	Myroxylon pereirae resin	1	1	1	0	1	0
**Benzocaine**	Benzocaine	0	0	0	1	0	1
**Benzyl alcohol**	Benzyl alcohol	0	0	1	1	0	0
**Benzyl salicylate**	Benzyl salicylate	0	0	0	1	1	0
**Black rubber mix**	N-Isopropyl-N'-phenyl-p-phenylenediamine	0	0	excluded†		n/a	n/a
	N-Cyclohexyl-N'-phenyl-p-phenylenediamine	0	0			n/a	n/a

	N,N′-Diphenyl-paraphenylenediamine	0	0			n/a	n/a
**Butylhydroxyanisole (BHA)**	Butylhydroxyanisole	1	1	1	1	n/a	n/a
**Bronopol**	Bronopol	1	1	1	0	n/a	n/a
**Cinnamal**	Cinnamal	1	1	1	1	1	1
**Cinnamyl alcohol**	Cinnamyl alcohol	1	1	1	1	1	1
**Citral**	Citral	0	1	1	1	1	1
**Cocamidopropyl betaine**	Cocamidopropyl betaine	1	1	0	0	0	0
**Colophonium**	Colophonium	1	1	1	1	n/a	n/a
**Diazolidynyl urea**	Diazolidynyl Urea	1	1	1	0	n/a	n/a
**DMDM Hydantoin**	DMDMD Hydantoin	1	1	1	0	n/a	n/a
**Epoxy resin**	Epichlorohydrin	0	0	0	0	n/a	n/a
	Isopropylidenediphenol	0	0	0	0	n/a	n/a
**Evernia prunastri extract**	Evernia prunastri extract	0	0	0	0	n/a	n/a
**Farnesol**	Farnesol	0	0	0	0	1	1
**Formaldehyde**	Formaldehyde	1	1	1	1	1	1
**Geraniol**	Geraniol	1	0	1	1	1	1
**Hydroxycitronellal**	Hydroxycitronellal	1	1	1	0	1	1
**Imidazolidynyl urea**	Imidazolidynyl urea	0	0	0	0	1	1
**Iodopropynyl butylcarbamate**	Iodopropynyl butylcarbamate	0	0	0	0	1	1
**Isopropyl myristate**	Isopropyl myristate	0	0	1	0	1	0
**Lanolin**	Lanolin	0	0	0	0	n/a	n/a
**Hydroxyisohexyl 3-cyclohexene carboxaldehyde (HICC) - Lyral**	Hydroxyisohexyl 3-cyclohexene carboxaldehyde	0	1	0	0	1	1
**Mercapto mix**	2,2′-Benzothiazyl disulfide	0	0	0	0	n/a	n/a
	4-Morpholinyl-2-benzothiazyl disulfide	0	0	0	0	n/a	n/a
	N-Cyclohexyl-2-benzothiazolesulfenamide	0	0	0	0	n/a	n/a
**Mercaptobenzothiazole**	Mercaptobenzothiazole	0	0	1	1	1	1
**Methylchloroisothiazolinone (MCI)**	Methylchloroisothiazolinone	1	1	1	0	1	1
**Methyldibromo Glutaronitrile (MDBGN)**	Methyldibromo Glutaronitrile	1	1	0	0	1	1
**Methylisothiazolinone (MI)**	Methylisothiazolinone	1	1	0	0	1	n/a

**Neomycin sulfate**	Neomycin sulfate	1	0	1	1	n/a	n/a
**Paraben mix**	Methylparaben	0	0	excluded†		0	1
	Butylparaben	0	0			n/a	n/a
	Ethylparaben	0	0			n/a	n/a
	Propylparaben	0	0			0	1
**p-Phenylenediamine (PPD)**	p-Phenylenediamine	1	0	1	1	1	1
**Propyl gallate**	Propyl gallate	0	0	1	1	1	1
**Quaternium 15**	Quaternium 15	1	1	1	1	n/a	n/a
**Quinoline mix**	Quinoline	0	0	0	0	n/a	n/a
	Chlorquinadol	0	0	0	0	n/a	n/a
**Sorbitan sesquioleate**	Sorbitol	0	0	0	1	n/a	n/a
	Oleic acid	0	0	0	0	n/a	n/a
**Thimerosal**	Thimerosal	1	0	1	1	n/a	n/a
**Triethanolamine**	Triethanolamine	0	0	1	1	n/a	n/a

†- multi-component ingredients with known sensitizing potential ("1") were excluded from the learning process, see explanations in text.

n/a – data not available

Table S5. Settings of WEKA classification algorithms tested during the model development process.

Algorithm	Algorithm settings
**Naïve Bayes classifier**	weka.classifiers.bayes.NaiveBayes
**Bayes nets**	weka.classifiers.bayes.BayesNet -D -Q weka.classifiers.bayes.net.search.local.K2 -- -P 1 -S BAYES -E weka.classifiers.bayes.net.estimate.SimpleEstimator -- -A 0.5
**Multilayer Perceptron**	weka.classifiers.functions.MultilayerPerceptron -L 0.3 -M 0.2 -N 500 -V 0 -S 0 -E 20 -H a
**SMO**	weka.classifiers.functions.SMO -C 1.0 -L 0.001 -P 1.0E-12 -N 0 -V -1 -W 1 -K "weka.classifiers.functions.supportVector.PolyKernel -E 1.0 -C 250007" -calibrator "weka.classifiers.functions.Logistic -R 1.0E-8 -M -1 -num-decimal-places
**kStar**	weka.classifiers.lazy.KStar -B 20 -M a
**RandomComittee**	weka.classifiers.meta.RandomCommittee -S 1 -num-slots 1 -I 10 -W weka.classifiers.trees.RandomTree -- -K 0 -M 1.0 -V 0.001 -S 1
**DecisionTable**	weka.classifiers.rules.DecisionTable -X 1 -S "weka.attributeSelection.BestFirst -D 1 -N 5"
**J48**	weka.classifiers.trees.J48 -C 0.25 -M 2

**RandomTree**	weka.classifiers.trees.RandomTree -K 0 -M 1.0 -V 0.001 -S 1
**RandomForest**	weka.classifiers.trees.RandomForest -P 100 -I 100 -num-slots 1 -K 0 -M 1.0 -V 0.001 -S 1

References:

1. An, S.; Lee, A.-Y.; Lee, C.H.; Kim, W.; Hahm, J.H.; Kim, K.-J.; Moon, K.-C.; Won, Y.H.; Ro, Y.-S.; Eun, H.C. Fragrance contact dermatitis in Korea: A joint study. *Contact Dermat.* **2005**, *53*, 320–323. https://doi.org/10.1111/j.0105-1873.2005.00720.x.
2. Heisterberg, M.V.; Menné, T.; Johansen, J.D. Contact allergy to the 26 specific fragrance ingredients to be declared on cosmetic products in accordance with the EU cosmetics directive. *Contact Dermat.* **2011**, *65*, 266–275. https://doi.org/10.1111/j.1600-0536.2011.01962.x.
3. Vejanurug, P.; Tresukosol, P.; Sajjachareonpong, P.; Puangpet, P. Fragrance allergy could be missed without patch testing with 26 individual fragrance allergens. *Contact Dermat.* **2016**, *74*, 230–235. https://doi.org/10.1111/cod.12522.
4. Wetter, D.A.; Yiannias, J.A.; Prakash, A.V.; Davis, M.D.; Farmer, S.A.; El-Azhary, R.A. Results of patch testing to personal care product allergens in a standard series and a supplemental cosmetic series: An analysis of 945 patients from the Mayo Clinic Contact Dermatitis Group, 2000-2007. *J. Am. Acad. Dermatol.* **2010**, *63*, 789–798. https://doi.org/10.1016/j.jaad.2009.11.033.
5. Diepgen, T.L.; Ofenloch, R.F.; Bruze, M.; Bertuccio, P.; Cazzaniga, S.; Coenraads, P.-J.; Elsner, P.; Goncalo, M.; Svensson, Å.; Naldi, L. Prevalence of contact allergy in the general population in different European regions. *Br. J. Dermatol.* **2016**, *174*, 319–329. https://doi.org/10.1111/bjd.14167.
6. Boyvat, A.; Akyol, A.; Gürgey, E. Contact sensitivity to preservatives in Turkey. *Contact Dermat.* **2005**, *52*, 329–332. https://doi.org/10.1111/j.0105-1873.2005.00607.x.

7. Larsen, W.; Nakayama, H.; Fischer, T.; Elsner, P.; Frosch, P.; Burrows, D.; Jordan, W.; Shaw, S.; Wilkinson, J.; Marks, J.; et al. Fragrance contact dermatitis: A worldwide multicenter investigation (Part II). *Contact Dermat.* **2001**, *44*, 344–346. https://doi.org/10.1034/j.1600-0536.2001.044006344.x.
8. Nardelli, A.; Carbonez, A.; Drieghe, J.; Goossens, A. Results of patch testing with fragrance mix 1, fragrance mix 2, and their ingredients, and*Myroxylon pereirae*and colophonium, over a 21-year period. *Contact Dermat.* **2013**, *68*, 307–313. https://doi.org/10.1111/cod.12056.
9. Kieć-Swierczyńska, M.; Krecisz, B.; Swierczyńska-Machura, D. Contact allergy to fragrances. *Med. Pr.* **2006**, *57*, 431–437.
10. Dinkloh, A.; Worm, M.; Geier, J.; Schnuch, A.; Wollenberg, A. Contact sensitization in patients with suspected cosmetic intolerance: Results of the IVDK 2006-2011. *J. Eur. Acad. Dermatol. Venereol.* **2015**, *29*, 1071–1081. https://doi.org/10.1111/jdv.12750.
11. Schnuch, A.; Uter, W.; Geier, J.; Lessmann, H.; Frosch, P.J. Sensitization to 26 fragrances to be labelled according to current European regulation. Results of the IVDK and review of the literature. *Contact Dermat.* **2007**, *57*, 1–10. https://doi.org/10.1111/j.1600-0536.2007.01088.x.
12. Uter, W.; Geier, J.; Frosch, P.; Schnuch, A. Contact allergy to fragrances: Current patch test results (2005-2008) from the Information Network of Departments of Dermatology*. *Contact Dermat.* **2010**, *63*, 254–261. https://doi.org/10.1111/j.1600-0536.2010.01759.x.
13. Larsen, W.; Nakayama, H.; Lindberg, M.; Fischer, T.; Elsner, P.; Burrows, D.; Jordan, W.; Shaw, S.; Wilkinson, J.; Marks, J., Jr.; et al. Fragrance contact dermatitis: A worldwide multicenter investigation (Part I). *Am. J. Contact Dermat.* **1996**, *7*, 77–83. https://doi.org/10.1016/S1046-199X(96)90078-0.
14. Krecisz, B.; Chomiczewska-Skóra, D.; Kieć-Świerczyńska, M. Preservatives as important etiologic factors of allergic contact dermatitis. *Med. Pr.* **2015**, *66*, 327–332. https://doi.org/10.13075/mp.5893.00176.
15. Dastychová, E.; Necas, M.; Vasku, V. Contact hypersensitivity to selected excipients of dermatological topical preparations and cosmetics in patients with chronic eczema. *Acta Dermatovenerol. Alp. Pannonica Adriat.* **2008**, *17*, 61–68.
16. Fairhurst, D.; Shah, M. Comparison of patch test results among white Europeans and patients from the Indian subcontinent living within the same community. *J. Eur. Acad. Dermatol. Venereol.* **2008**, *22*, 1227–1231. https://doi.org/10.1111/j.1468-3083.2008.02787.x.
17. Schnuch, A.; Geier, J.; Uter, W.; Frosch, P.J. Patch testing with preservatives, antimicrobials and industrial biocides. Results from a multicentre study. *Br. J. Dermatol.* **1998**, *138*, 467–476. https://doi.org/10.1046/j.1365-2133.1998.02126.x.
18. Herbst, R.A.; Uter, W.; Pirker, C.; Geier, J.; Frosch, P.J. Allergic and non-allergic periorbital dermatitis: Patch test results of the Information Network of the Departments of Dermatology during a 5-year period. *Contact Dermat.* **2004**, *51*, 13–19. https://doi.org/10.1111/j.0105-1873.2004.00334.x.
19. Davis, M.D.P.; Scalf, L.A.; Yiannias, J.A.; Cheng, J.F.; El-Azhary, R.A.; Rohlinger, A.L.; Farmer, S.A.; Fett, D.D.; Johnson, J.S.; Linehan, D.L.N.; et al. Changing Trends and Allergens in the Patch Test Standard Series: A mayo clinic 5-year retrospective review. *Arch. Dermatol.* **2008**, *144*, 67–72. https://doi.org/10.1001/archdermatol.2007.2.
20. Perrenoud, D.; Bircher, A.; Hunziker, T.; Sutter, H.; Bruckner-Tuderman, L.; Stäger, J.; Thürlimann, W.; Schmid, P.; Suard, A.; Swiss Contact Dermatitis Research Group. Frequency of sensitization to 13 common preservatives in Switzerland. *Contact Dermat.* **1994**, *30*, 276–279. https://doi.org/10.1111/j.1600-0536.1994.tb00597.x.
21. O'Gorman, S.M.; Torgerson, R.R. Contact allergy in cheilitis. *Int. J. Dermatol.* **2015**, *55*, e386–e391. https://doi.org/10.1111/ijd.13044.
22. Lee, S.S.; Hong, D.K.; Jeong, N.J.; Lee, J.H.; Choi, Y.; Lee, A.-Y.; Lee, C.-H.; Kim, K.J.; Park, H.Y.; Yang, J.; et al. Multicenter study of preservative sensitivity in patients with suspected cosmetic contact dermatitis in Korea. *J. Dermatol.* **2012**, *39*, 677–681. https://doi.org/10.1111/j.1346-8138.2012.01551.x.
23. Rodrigues, D.F.; Goulart, E.M.A. Patch test results in children and adolescents. Study from the Santa Casa de Belo Horizonte Dermatology Clinic, Brazil, from 2003 to 2010. *An. Bras. Dermatol.* **2015**, *90*, 671–683. https://doi.org/10.1590/abd1806-4841.20153902.

24. Schwensen, J.F.; Johansen, J.D.; Veien, N.K.; Funding, A.T.; Avnstorp, C.; Østerballe, M.; Andersen, K.E.; Paulsen, E.; Mortz, C.G.; Sommerlund, M.; et al. Occupational contact dermatitis in hairdressers: An analysis of patch test data from the Danish Contact Dermatitis Group, 2002–2011. *Contact Dermat.* **2013**, *70*, 233–237. https://doi.org/10.1111/cod.12176.
25. Schwensen, J.F.; Menné, T.; Veien, N.K.; Funding, A.T.; Avnstorp, C.; Østerballe, M.; Andersen, K.E.; Paulsen, E.; Mortz, C.G.; Sommerlund, M.; et al. Occupational contact dermatitis in blue-collar workers: Results from a multicentre study from the Danish Contact Dermatitis Group (2003–2012). *Contact Dermat.* **2014**, *71*, 348–355. https://doi.org/10.1111/cod.12277.
26. Sharma, V.K.; Chakrabarti, A. Common contact sensitizers in Chandigarh, India. A study of 200 patients with the European standard series. *Contact Dermat.* **1998**, *38*, 127–131. https://doi.org/10.1111/j.1600-0536.1998.tb05677.x.
27. Yin, R.; Huang, X.Y.; Zhou, X.F.; Hao, F. A retrospective study of patch tests in Chongqing, China from 2004 to 2009. *Contact Dermat.* **2011**, *65*, 28–33. https://doi.org/10.1111/j.1600-0536.2010.01854.x.
28. Schnuch, A.; Lessmann, H.; Geier, J.; Uter, W. Contact allergy to preservatives. Analysis of IVDK data 1996–2009. *Br. J. Dermatol.* **2011**, *164*, 1316–1325. https://doi.org/10.1111/j.1365-2133.2011.10253.x.
29. Chow, E.T.; Avolio, A.M.; Lee, A.; Nixon, R. Frequency of positive patch test reactions to preservatives: The Australian experience. *Australas. J. Dermatol.* **2012**, *54*, 31–35. https://doi.org/10.1111/j.1440-0960.2012.00958.x.
30. Uter, W.; Gefeller, O.; John, S.M.; Schnuch, A.; Geier, J. Contact allergy to ingredients of hair cosmetics—A comparison of female hairdressers and clients based on IVDK 2007–2012 data. *Contact Dermat.* **2014**, *71*, 13–20. https://doi.org/10.1111/cod.12196.
31. Cuesta, L.; Silvestre, J.F.; Toledo, F.; Lucas, A.; Ballester, I.; Pérez-Crespo, M.; Ballester, I. Fragrance contact allergy: A 4-year retrospective study. *Contact Dermat.* **2010**, *63*, 77–84. https://doi.org/10.1111/j.1600-0536.2010.01739.x.
32. Fall, S.; Bruze, M.; Isaksson, M.; Lidén, C.; Matura, M.; Stenberg, B.; Lindberg, M. Contact allergy trends in Sweden—A retrospective comparison of patch test data from 1992, 2000, and 2009. *Contact Dermat.* **2015**, *72*, 297–304. https://doi.org/10.1111/cod.12346.
33. Lestringant, G.G.; Bener, A.; Sawaya, M.; Galadari, I.H.; Frossard, P.M. Allergic contact dermatitis in the United Arab Emirates. *Int. J. Dermatol.* **1999**, *38*, 181–186. https://doi.org/10.1046/j.1365-4362.1999.00628.x.
34. Li, L.-F. Contact sensitization to European baseline series of allergens in university students in Beijing. *Contact Dermat.* **2010**, *62*, 371–372. https://doi.org/10.1111/j.1600-0536.2010.01723.x.
35. Schwensen, J.; Menné, T.; Sommerlund, M.; Andersen, K.; Mortz, C.; Zachariae, C.; Johansen, J. Contact Allergy in Danish Healthcare Workers: A Retrospective Matched Case-control Study. *Acta Derm. Venereol.* **2016**, *96*, 237–240. https://doi.org/10.2340/00015555-2202.
36. Frosch, P.J.; Pirker, C.; Rastogi, S.C.; Andersen, K.E.; Bruze, M.; Svedman, C.; Goossens, A.; White, I.R.; Uter, W.; Arnau, E.G.; et al. Patch testing with a new fragrance mix detects additional patients sensitive to perfumes and missed by the current fragrance mix. *Contact Dermat.* **2005**, *52*, 207–215. https://doi.org/10.1111/j.0105-1873.2005.00565.x.
37. Molin, S.; Bauer, A.; Schnuch, A.; Geier, J. Occupational contact allergy in nurses: Results from the Information Network of Departments of Dermatology 2003–2012. *Contact Dermat.* **2014**, *72*, 164–171. https://doi.org/10.1111/cod.12330.
38. Thyssen, J.P.; Engkilde, K.; Lundov, M.D.; Carlsen, B.C.; Menné, T.; Johansen, J.D. Temporal trends of preservative allergy in Denmark (1985–2008). *Contact Dermat.* **2010**, *62*, 102–108. https://doi.org/10.1111/j.1600-0536.2009.01668.x.

39. Britton, J.; Wilkinson, S.; English, J.; Gawkrodger, D.; Ormerod, A.; Sansom, J.; Shaw, S.; Statham, B. The British standard series of contact dermatitis allergens: Validation in clinical practice and value for clinical governance. *Br. J. Dermatol.* **2003**, *148*, 259–264. https://doi.org/10.1046/j.1365-2133.2003.05170.x.
40. Ford, G.P.; Beck, M.H. Reactions to Quaternium 15, Bronopol and Germall 115 in a standard series. *Contact Dermat.* **1986**, *14*, 271–274. https://doi.org/10.1111/j.1600-0536.1986.tb05274.x.
41. Jong, C.T.; Statham, B.N.; Green, C.M.; King, C.M.; Gawkrodger, D.J.; Sansom, J.E.; English, J.S.C.; Wilkinson, S.M.; Ormerod, A.D.; Chowdhury, M. Contact sensitivity to preservatives in the UK, 2004?2005: Results of multicentre study. *Contact Dermat.* **2007**, *57*, 165–168. https://doi.org/10.1111/j.1600-0536.2007.01181.x.
42. Shaughnessy, C.N.; Malajian, D.; Belsito, D.V. Cutaneous delayed-type hypersensitivity in patients with atopic dermatitis: Reactivity to topical preservatives. *J. Am. Acad. Dermatol.* **2014**, *70*, 102–107. https://doi.org/10.1016/j.jaad.2013.08.046.
43. Katsarma, G.; Gawkrodger, D.J. Suspected fragrance allergy requires extended patch testing to individual fragrance allergens. *Contact Dermat.* **1999**, *41*, 193–197. https://doi.org/10.1111/j.1600-0536.1999.tb06129.x.
44. Larsen, W.; Nakayama, H.; Fischer, T.; Elsner, P.; Frosch, P.; Burrows, D.; Jordan, W.; Shaw, S.; Wilkinson, J.; Marks, J.; et al. Fragrance contact dermatitis—A worldwide multicenter investigation (Part III). *Contact Dermat.* **2002**, *46*, 141–144. https://doi.org/10.1034/j.1600-0536.2002.460302.x.
45. Tomar, J.; Jain, V.K.; Aggarwal, K.; Dayal, S.; Gupta, S. Contact Allergies to Cosmetics: Testing with 52 Cosmetic Ingredients and Personal Products. *J. Dermatol.* **2005**, *32*, 951–955. https://doi.org/10.1111/j.1346-8138.2005.tb00880.x.
46. Spiewak, R.; Samochocki, Z.; Grubska-Suchanek, E.; Czarnobilska, E.; Pasnicki, M.; Czarnecka-Operacz, M.; Bukiel, M.; Cisowska, A.; Jedrzejewska-Jurga, K.; Krakowski, A.; et al. Gallates. as well as hydroperoxides of limonene and linalol are more frequent and relevant sensitizers than any cosmetic ingredient included in the European Baseline Series. *Contact Dermat.* **2016**, *75*, 87.
47. Ochi, H.; Cheng, S.W.N.; Leow, Y.H.; Goon, A.T.J. Contact allergy trends in Singapore—A retrospective study of patch test data from 2009 to 2013. *Contact Dermat.* **2016**, *76*, 49–50. https://doi.org/10.1111/cod.12629.
48. Frosch, P.J.; Rastogi, S.C.; Pirker, C.; Brinkmeier, T.; Andersen, K.E.; Bruze, M.; Svedman, C.; Goossens, A.; White, I.R.; Uter, W.; et al. Patch testing with a new fragrance mix—Reactivity to the individual constituents and chemical detection in relevant cosmetic products. *Contact Dermat.* **2005**, *52*, 216–225. https://doi.org/10.1111/j.0105-1873.2005.00563.x.
49. Isaksson, M.; Inerot, A.; Lidén, C.; Lindberg, M.; Matura, M.; Möller, H.; Stenberg, B.; Bruze, M. Multicentre patch testing with fragrance mix II and hydroxyisohexyl 3-cyclohexene carboxaldehyde by the Swedish Contact Dermatitis Research Group. *Contact Dermat.* **2014**, *70*, 187–189. https://doi.org/10.1111/cod.12156.
50. Krautheim, A.; Uter, W.; Frosch, P.; Schnuch, A.; Geier, J. Patch testing with fragrance mix II: Results of the IVDK 2005–2008. *Contact Dermat.* **2010**, *63*, 262–269. https://doi.org/10.1111/j.1600-0536.2010.01753.x.
51. Ito, A.; Nishioka, K.; Kanto, H.; Yagami, A.; Yamada, S.; Sugiura, M.; Yasunaga, C.; Yoshii, K.; Kobayashi, H.; Adachi, A.; et al. A multi-institutional joint study of contact dermatitis related to hair colouring and perming agents in Japan. *Contact Dermat.* **2017**, *77*, 42–48. https://doi.org/10.1111/cod.12783.
52. Thyssen, J.; Linneberg, A.; Menné, T.; Nielsen, N.; Johansen, J. Contact allergy to allergens of the TRUE-test (panels 1 and 2) has decreased modestly in the general population. *Br. J. Dermatol.* **2009**, *161*, 1124–1129. https://doi.org/10.1111/j.1365-2133.2009.09325.x.
53. Dotterud, L.K.; Smith-Sivertsen, T. Allergic contact sensitization in the general adult population: A population-based study from Northern Norway. *Contact Dermat.* **2007**, *56*, 10–15. https://doi.org/10.1111/j.1600-0536.2007.00980.x.

54. Nielsen, N.H.; Menné, T. Allergic contact sensitization in an unselected Danish population. The Glostrup Allergy Study, Denmark. *Acta Derm. Venereol.* **1992**, *72*, 456–460.
55. Nielsen, N.H.; Linneberg, A.; Menné, T.; Madsen, F.; Frølund, L.; Dirksen, A.; Jørgensen, T. Allergic Contact Sensitization in an Adult Danish Population: Two Cross-sectional Surveys Eight Years Apart (The Copenhagen Allergy Study). *Acta Derm. Venereol.* **2001**, *81*, 31–34. https://doi.org/10.1080/000155501750208155.
56. Schäfer, T.; Böhler, E.; Ruhdorfer, S.; Weigl, L.; Wessner, D.; Filipiak, B.; Wichmann, H.E.; Ring, J. Epidemiology of contact allergy in adults. *Allergy* **2001**, *56*, 1192–1196. https://doi.org/10.1034/j.1398-9995.2001.00086.x.
57. Zhao, J.; Li, L.-F. Contact sensitization to cosmetic series of allergens in a general population in Beijing. *J. Cosmet. Dermatol.* **2014**, *13*, 68–71. https://doi.org/10.1111/jocd.12078.
58. White, J.M.L.; Gilmour, N.J.; Jeffries, D.; Duangdeeden, I.; Kullavanijaya, P.; Basketter, D.A.; McFadden, J.P. A general population from Thailand: Incidence of common allergens with emphasis on para-phenylenediamine. *Clin. Exp. Allergy* **2007**, *37*, 1848–1853. https://doi.org/10.1111/j.1365-2222.2007.02846.x.
